# Mitochondrial dysfunction promotes the transition of precursor to terminally exhausted T cells through HIF-1α-mediated glycolytic reprogramming

**DOI:** 10.1038/s41467-023-42634-3

**Published:** 2023-10-27

**Authors:** Hao Wu, Xiufeng Zhao, Sophia M. Hochrein, Miriam Eckstein, Gabriela F. Gubert, Konrad Knöpper, Ana Maria Mansilla, Arman Öner, Remi Doucet-Ladevèze, Werner Schmitz, Bart Ghesquière, Sebastian Theurich, Jan Dudek, Georg Gasteiger, Alma Zernecke, Sebastian Kobold, Wolfgang Kastenmüller, Martin Vaeth

**Affiliations:** 1 Würzburg Institute of Systems Immunology, Max Planck Research Group, Julius-Maximilians University of Würzburg, Würzburg, Germany; 2 Division of Clinical Pharmacology, Department of Medicine IV, Ludwig Maximilians University (LMU) Munich, University Hospital, Munich, Germany; 3 Department of Biochemistry and Molecular Biology, Theodor Boveri Institute, Biocenter, Julius-Maximilians University of Würzburg, Würzburg, Germany; 4 Laboratory of Applied Mass Spectrometry, Department of Cellular and Molecular Medicine, KU Leuven, Leuven, Belgium and Metabolomics Core Facility Leuven, Center for Cancer Biology, VIB, Leuven, Belgium; 5 Ludwig Maximilians University (LMU) Munich, University Hospital, Department of Medicine III, Munich, Germany and LMU Gene Center, Cancer and Immunometabolism Research Group, Munich, Germany; 6 German Cancer Consortium (DKTK), partner site Munich, a partnership between the DKFZ Heidelberg and the University Hospital of the LMU, Munich, Germany; 7 Comprehensive Heart Failure Center (CHFC), University Hospital, Julius-Maximilians University of Würzburg, Würzburg, Germany; 8 Institute of Experimental Biomedicine, University Hospital Würzburg, Würzburg, Germany

**Keywords:** Lymphocyte differentiation, Infection, Translational research, Cytotoxic T cells

## Abstract

T cell exhaustion is a hallmark of cancer and persistent infections, marked by inhibitory receptor upregulation, diminished cytokine secretion, and impaired cytolytic activity. Terminally exhausted T cells are steadily replenished by a precursor population (Tpex), but the metabolic principles governing Tpex maintenance and the regulatory circuits that control their exhaustion remain incompletely understood. Using a combination of gene-deficient mice, single-cell transcriptomics, and metabolomic analyses, we show that mitochondrial insufficiency is a cell-intrinsic trigger that initiates the functional exhaustion of T cells. At the molecular level, we find that mitochondrial dysfunction causes redox stress, which inhibits the proteasomal degradation of hypoxia-inducible factor 1α (HIF-1α) and promotes the transcriptional and metabolic reprogramming of Tpex cells into terminally exhausted T cells. Our findings also bear clinical significance, as metabolic engineering of chimeric antigen receptor (CAR) T cells is a promising strategy to enhance the stemness and functionality of Tpex cells for cancer immunotherapy.

## Abstract (web summary)

Exhaustion is the functional deterioration of T cells following chronic stimulation. Here, Wu et al. show that mitochondrial dysfunction drives T cell exhaustion by inhibiting HIF-1α degradation and transcriptional metabolic reprogramming.

## Introduction

Following an acute infection, CD8^+^ T cells proliferate and differentiate into cytotoxic T lymphocytes (CTLs) to eliminate infected target cells. The differentiation of CTLs is paralleled by transcriptional, epigenetic, and metabolic rewiring to support their clonal expansion and effector functions, such as cytokine secretion and cytotoxicity^1,2^. After the clearance of the pathogen and the resolution of inflammation, most effector T cells die and only a few lymphocytes remain as long-lived memory T cells.

However, when antigens cannot be eliminated, such as in persistent infection and cancer, continuous antigen receptor stimulation drives an alternative differentiation trajectory, known as T cell exhaustion^1–3^. Exhausted T (Tex) cells are characterized phenotypically by the upregulation of co-inhibitory receptors, such as PD-1, Tim-3, and Lag-3, and functionally by the decline in cytokine secretion and cytolytic activity^1,3^. Although Tex cells lose proliferative capacity and undergo apoptosis during terminal differentiation, they are steadily replenished by a distinct multipotent population, termed precursors (or progenitors) of exhausted T (Tpex) cells^3–7^. Tpex cells can be identified by the expression of memory-related molecules, such as the transcription factor TCF-1 and the cell surface molecules Ly108 and CD62L, but the absence of terminal differentiation markers, such as Tim-3 and CD160^3,4,7–10^. The ’stem-like’ capacity of Tpex cells to self-renew and their superior repopulation potential following adoptive transfer into chronically infected mice has spurred increasing scientific interest to define the molecular mechanisms that control the transition from multipotent Tpex into dysfunctional Tex cells^3,6^. This question is also clinically relevant since Tpex cells are the main mediators of the antitumor immune responses following therapeutic checkpoint blockade^3,5,6,8,11^. Although PD-1 is expressed on both Tpex and terminally exhausted T cells, only Tpex cells respond efficiently to anti-PD-1 treatment with a proliferative burst and the differentiation into cytotoxic T cells, thus reinvigorating antitumor immune responses in patients following checkpoint immunotherapy^3,5,6,11,12^. However, the molecular mechanisms that maintain the stemness of Tpex cells and the signals that instruct their functional exhaustion remain incompletely understood and merit further investigation to improve the clinical outcome of cancer immunotherapy.

Naïve T cells are characterized by a low-rate catabolic metabolism and generate ATP primarily through mitochondrial respiration and fatty acid oxidation (FAO)^13,14^. Antigen receptor ligation triggers a Ca^2+^ influx cascade in T cells, which activates the serine/threonine phosphatase calcineurin promoting the nuclear translocation of *nuclear factor of activated T cells* (NFAT)^15,16^. In cooperation with other transcription factors, NFAT controls the expression of nutrient transporters and metabolic enzymes to support the cellular growth and clonal expansion of activated T cells^17–19^. To meet their increased bioenergetic demand, activated T cells rewire their metabolic machinery and utilize aerobic glycolysis and oxidative phosphorylation (OXPHOS) for ATP production and the biosynthesis of building blocks^14,17,18^. Although metabolic reprogramming controls multiple aspects of T cell activation and effector/memory differentiation^13,14^, the regulation of metabolism and its contribution to T cell dysfunction is not fully understood^2^. Recent studies have demonstrated that mitochondrial decline correlates with T cell exhaustion in vitro^20,21^, during chronic viral infection^22,23^ and in cancer^24,25^, but whether these changes are causes or consequences of T cell exhaustion remained unclear.

Here, we provide genetic evidence that impaired mitochondrial respiration is not merely a consequence of T cell dysfunction but, instead, is sufficient to elicit the transcriptional, phenotypic, and functional hallmarks of T cell exhaustion. Mechanistically, we found that oxidative stress due to mitochondrial deterioration antagonizes the proteasomal degradation of *hypoxia-inducible factor 1* α (HIF-1α), which mediates the glycolytic reprogramming of Tpex cells as an initial step towards terminal differentiation. Our findings also demonstrate that mitochondrial respiration is a prerequisite for the stemness of exhausted T cells and that limiting their glycolytic capacity is a promising metabolic strategy to maintain the functionality of T cells during chronic viral infection and cancer.

## Results

### Terminal T cell exhaustion is characterized by metabolic reprogramming

**Fig. 1:**
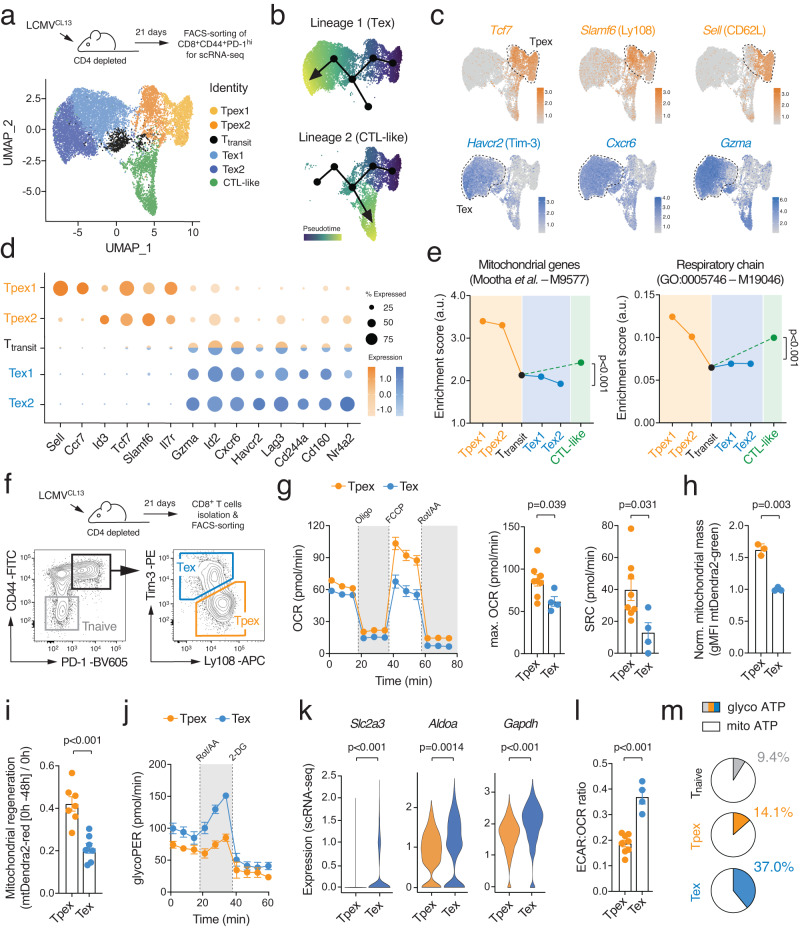
T cell exhaustion is characterized by metabolic reprogramming. **a** C57BL/6 mice were chronically infected with the LCMV strain clone 13 (LCMV^CL13^) and CD8^+^CD44^+^PD-1^hi^ T cells were subjected to single-cell RNA sequencing 21 days post infection. Uniform manifold approximation and projection (UMAP) visualization of ~ 13.000 non-proliferating (*Mki67* negative) T cells colored based on their classification into six clusters. **b** Prediction of developmental trajectories using slingshot analysis; cells are color-coded according to pseudotime. **c** Normalized gene expression of *Tcf7*, *Slamf6* (Ly108), *Sell* (CD62L), *Havcr2* (Tim-3), *Cxcr6* and *Gzma* projected onto UMAP clusters. **d** Dot plot analysis of selected markers representing Tpex and Tex cell subsets; color intensity and dot size represent z-score mean expression and percentage of cells expressing the gene, respectively. **e** Enrichment of mitochondrial and respiratory chain gene expression signatures in clusters representing Tpex, CTL-like and terminally exhausted (Tex) cells. **f** Flow cytometric cell sorting strategy of Tpex and Tex cells from chronically infected mice. **g** Analyzes of oxygen consumption rate (OCR) and spare respiratory capacity (SRC) of Tpex (*n* = 9 mice) and Tex cells (*n* = 4 mice) using a Seahorse extracellular flux analyzer; means ± SEM. **h** Analysis of mitochondrial content in Tpex and Tex cells using LCMV^CL13^ infected mito-Dendra2 mice; means ± SEM of 3 mice. **i** Mitochondrial regeneration of mito-Dendra2 Tpex and Tex cells after photoconversion in vitro; means ± SEM of 7 mice. **j** Glycolytic proton efflux rate (glycoPER) analyzes of Tpex (*n* = 5 mice) and Tex cells (*n* = 2 mice) using a Seahorse extracellular flux analyzer; means ± SEM. **k** Violin plots displaying *Slc2a3* (GLUT3), *Aldoa* and *Gapdh* gene expression in Tpex and Tex cell clusters as shown in (**a**). **l** Ratio of extracellular acidification rate (ECAR) to OCR of FACS-sorted Ly108^+^ Tpex and Tim3^+^ Tex isolated from chronically infected mice ex vivo; means ± SEM of 4-8 mice. **m** Relative contribution of glycolysis and mitochondrial respiration to cellular ATP production in naïve (*n* = 10 mice), Tpex (*n* = 6 mice) and Tex cells (*n* = 4 mice). Unpaired two-tailed Student’s t-test in (**g**-**i**), (**k**), (**l**) and two-way ANOVA in (**e**).

To analyze the transcriptional-metabolic programs along the developmental trajectories of exhausted T cells, we performed single-cell (sc) RNA sequencing of CD44^+^ PD-1^hi^ CD8^+^ T cells isolated from chronically infected wild-type (WT) mice after infection with the clone 13 strain of lymphocytic choriomeningitis virus (LCMV^CL13^) (Supplementary Fig. 1a). To avoid a bias due to active cell division, we focused on non-proliferating T cells and identified six major populations of exhausted T cells using unsupervised clustering visualized by uniform manifold approximation and projection (UMAP) (Fig. 1a and Supplementary Fig. 1b). Tpex cells were characterized by a unique combination of activation (*Cd44, Cd69, Icos*), exhaustion (*Pdcd1*, *Tox*) and memory-related molecules (*Sell, Ccr7, Tcf7, Slamf6, Id3*, *Cxcr5*) (Fig. 1b-d and Supplementary Fig. 1b, c). Diffusion pseudotime analysis predicted two differentiation trajectories originating from ‘stem-like’ CD62L^+^ Tpex1 cells^9^ (Fig. 1b). One trajectory merged into a ‘CTL-like’ exhausted T cell population that is characterized by *Cx3cr1*, *Klrg1* and *S100a4* expression (Supplementary Fig. 1c)^26^. The second lineage pointed towards terminally exhausted T cells (Fig. 1b). Indeed, the Tex1 and Tex2 subsets showed a progressive upregulation of terminal exhaustion markers, such as *Havcr2* (encoding for Tim-3), *Cxcr6*, *Id2*, *Lag3*, *Gzma*, *Cd244a* (2B4) and *Tnfrsf9* (4-1BB) (Fig. 1c, d and Supplementary Fig. 1c). The bifurcation of the two lineages occurred in the small, but distinct, T_transit_ cluster that is marked by the downregulation of Tpex-associated genes, including *Sell* (CD62L), *Ccr7*, *Tcf7*, *Il7r*, *Id3*, and *Slamf6* (Ly108), and an intermediate expression of terminal exhaustion markers (Fig. 1d). To further explore the metabolic-transcriptional programs during the differentiation of stem-like CD62L^+^ Tpex cells into CTL-like and terminally exhausted T cells, we calculated gene set enrichment scores for metabolic pathways, such as glycolysis, mitochondrial respiration, lipid metabolism and the pentose phosphate pathway (Supplementary Table S1). These analyzes revealed a sharp decline in mitochondrial (gene set ID M9577) and respiratory chain gene expression (M19046) at the transition between Tpex to terminally differentiated T cells (Fig. 1e), indicating a relationship between mitochondrial (dys-) function and T cell exhaustion. In parallel to our scRNA sequencing data, we analyzed publicly available bulk RNA sequencing data of ID3^+^ Tpex and Tim-3^+^ Tex cells from chronically infected mice^4^ (Supplementary Fig. 1d, e). Gene set enrichment analyzes (GSEA) and clustering of gene networks revealed a significant correlation of pathways involved in *mitochondrial metabolism* and *translation* with Tpex cells, whereas the transcriptomes of Tex cells were primarily associated with *signal transduction, cell cycle* and *DNA repair* gene expression signatures (Supplementary Fig. 1d). The notion that Tpex cells rely more on mitochondrial respiration than Tex cells was further supported by GSEA using gene ontology (GO) pathways, such as *mitochondrial genes* (M9577), *mitochondrial translation* (M27446) and *respiratory electron transport and ATP synthesis by chemiosmotic coupling* (M1025) (Supplementary Fig. 1e).

To directly measure mitochondrial respiration in exhausted T cells, we infected WT mice with LCMV^CL13^ and isolated Ly108^+^ Tpex and Tim-3^+^ Tex cells by FACS sorting (Fig. 1f). In line with the transcriptomic data, extracellular flux analyzes revealed higher basal and maximal oxygen consumption rates (OCR) of Tpex compared to Tex cells (Fig. 1g), indicating that Tpex cells are more reliant on OXPHOS. The spare respiratory capacity (SRC), calculated as the difference between maximal and basal OCR, suggested that Tpex cells have a greater metabolic reserve to utilize mitochondrial respiration, whereas Tex cells operate OXPHOS almost at their maximal capacity (Fig. 1g). The difference in the SRC can be explained by an altered mitochondrial content and/or differences in their mitochondrial regenerative capacity. To test these possibilities, we employed mito-Dendra2 mice^27^, which express a mitochondrial-localized version of the Dendra2 protein. Leveraging the green fluorescence of mito-Dendra2, which correlates with the mass/volume of the mitochondria^27^, we could readily detect that the mitochondrial content of Tpex cells was significantly higher compared to Tex cells (Fig. 1h and Supplementary Fig. 2a). To delineate mitochondrial turnover and regeneration capacity in exhausted T cells, we labeled T cells of chronically infected mito-Dendra2 mice with cell trace violet (CTV) and irreversibly photo-switched their mitochondrial fluorescence emission spectrum from green to red fluorescence upon exposure to 405 nm laser light (Supplementary Fig. 2a, b). We tracked the emergence of non-red mitochondria in undivided Tpex and Tex cells in vitro as a measure for mitochondrial regeneration (Fig. 1i and Supplementary Fig. 2b). Mitotracker and TMRE probes validated lower mitochondrial content and membrane potential in Tex compared to Tpex cells (Supplementary Fig. 2c). Furthermore, Tex cells showed a diminished expression of electron transport chain (ETC) complexes (Supplementary Fig. 2d, e) and mitochondrial biogenesis factors, such as PGC-1α and TFAM (Supplementary Fig. 2d).

**Fig. 2:**
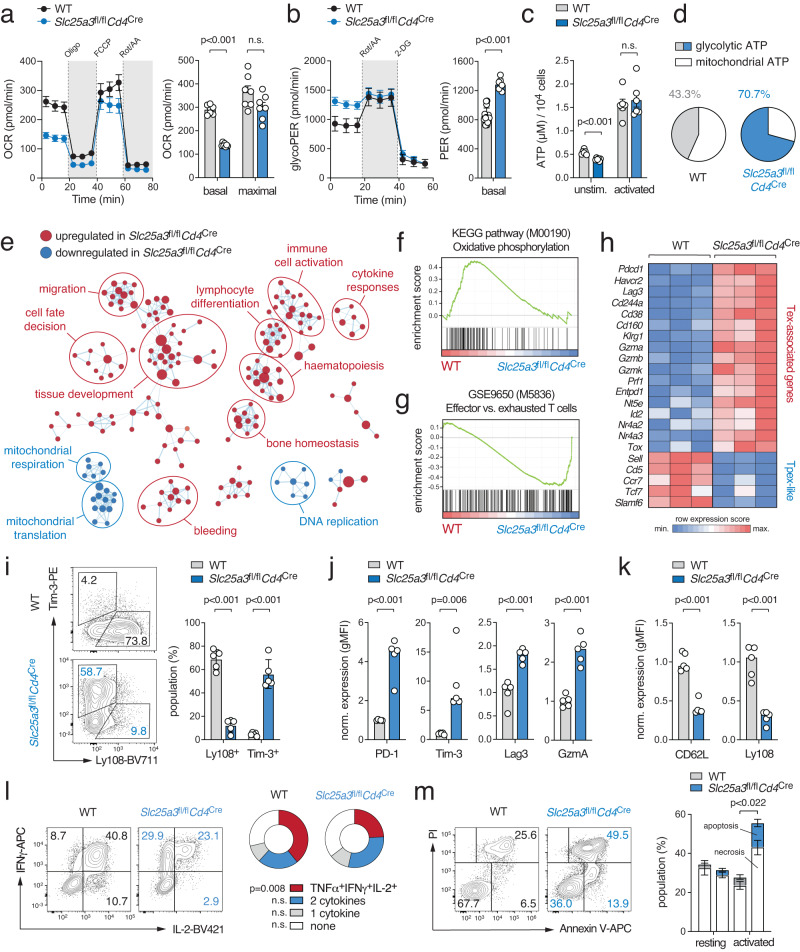
Genetic suppression of mitochondrial ATP production promotes glycolytic-transcriptional reprogramming of T cells. **a**, **b** Analyzes of (**a**) oxygen consumption rate (OCR) and (**b**) glycolytic proton efflux rate (glycoPER) of WT and mPiC-deficient (*Slc25a3*^fl/fl^*Cd4*^Cre^) T cells at day 2 of culture using a Seahorse extracellular flux analyzer; means ± SEM of 7 mice. **c** Analysis of ATP concentrations in unstimulated and anti-CD3/CD28 activated WT and mPiC-deficient T cells; means ± SEM of 6 mice. **d** Relative contribution of glycolysis and mitochondrial respiration to cellular ATP production in anti-CD3/CD28 stimulated WT and mPiC-deficient T cells; means of 3 mice. **e** Network clustering of RNA sequencing data of significantly (*p* < 0.05) enriched gene expression signatures between CTL-differentiated WT and mPiC-deficient T cells. Down- and upregulated gene signatures in mPiC-deficient T cells are shown in blue and red, respectively. **f**, **g** Gene set enrichment analysis (GSEA) of (**f**) *oxidative phosphorylation* (KEGG pathway) and (**g**) *effector versus exhausted T cells* (GSE9650) gene signatures in differentiated WT and mPiC-deficient T cells after 6 days of culture. **h** Heatmap expression analysis of selected genes in WT and mPiC-deficient CTLs after 6 days in culture. **i**–**k** Differentiation of WT and mPiC-deficient T cells in vitro, means ± SEM of 5 mice. **j**, **k** Flow cytometric quantification of (**j**) exhaustion and (**k**) memory marker expression in WT and mPiC-deficient CTLs, means ± SEM of 5 mice. **l** Analysis of polyfunctional TNFα, IFNγ and IL-2 expression by WT and mPiC-deficient T cells after 6 days of culture and anti-CD3/CD28 restimulation; means ± SEM of 3 mice. **m** Analysis of apoptosis in resting and anti-CD3/CD28 stimulated T cells by flow cytometry; means ± SEM of 3 mice. Two-tailed unpaired Student’s t-test in (**a**–**c**) and (**i**–**m**).

In contrast to their impaired mitochondrial performance, Tex cells showed an elevated glycolytic proton efflux rate (glycoPER) compared to Tpex cells (Fig. 1j), suggesting that Tex cells compensate their mitochondrial insufficiency by elevated aerobic glycolysis. The increased extracellular acidification rate (ECAR) of Tex cells correlated with a greater expression of nutrient transporters and glycolytic enzymes, such as *Slc2a3* (encoding GLUT3^28^), *Aldoa* and *Gapdh* (Fig. 1k). The notion that T cell exhaustion is mediated by a metabolic switch from mitochondrial respiration to aerobic glycolysis was further supported by a higher ECAR:OCR ratio in Tex cells compared to precursor cells (Fig. 1l). When we calculated the contribution of glycolytic and mitochondrial metabolism to ATP production, we found that Tpex cells rely primarily on OXPHOS, whereas Tex cells showed a ~ 2.5-fold greater dependency on aerobic glycolysis (Fig. 1m). Collectively, these data show that diminished mitochondrial respiration and a Warburg effect-like glycolytic reprogramming is a metabolic hallmark of terminal T cell exhaustion.

### Mitochondrial respiration controls the functional exhaustion of T cells

Having established that impaired mitochondrial ATP production coincides with the transition of Tpex into Tex cells, we set out to delineate if these metabolic changes are a consequence or the cause of T cell exhaustion. To directly address the role of mitochondrial bioenergetics, we generated mice with T cell-specific deletion of the *Slc25a3* gene that encodes for the mitochondrial phosphate carrier (mPiC)^29^. Inactivation of mPiC causes a paucity of free inorganic phosphate within the mitochondrial matrix as a rate-limiting step in the biosynthesis of mitochondrial ATP (Supplementary Fig. 3a)^29^. Thus, *Slc25a3*^fl/fl^*Cd4*^Cre^ mice are an excellent genetic model to investigate the causality between mitochondrial ATP production and T cell exhaustion. Mice with T cell-specific mPiC ablation were born at the expected Mendelian ratio without obvious defects in the thymic T cell development (Supplementary Fig. 3b, c). However, *Slc25a3*^fl/fl^*Cd4*^Cre^ mice had reduced conventional (Supplementary Fig. 3d) and regulatory T cells (Supplementary Fig. 3e) in their peripheral lymphoid organs. The phenotype of the CD4^+^ and CD8^+^ T cells in *Slc25a3*^fl/fl^*Cd4*^Cre^ mice was slightly shifted towards CD44^+^CD62L^–^ effector T cells at the expense of naïve and CD44^+^CD62L^+^ central memory cells (Supplementary Fig. 3f, g). To examine the metabolic profiles of mPiC-deficient T cells, we stimulated naive CD8^+^ T cells of with anti-CD3/CD28 for 2 days (activation) followed by an incubation with IL-2 for additional 4 days (CTL differentiation) (Fig. 2a–d and Supplementary Fig. 3h, i). Seahorse extracellular flux analyzes revealed markedly reduced basal and maximal OCR levels in anti-CD3/CD28 activated mPiC-deficient T cells (Fig. 2a) coinciding with higher glycolytic activity (Fig. 2b). A similar metabolic phenotype was also observed in fully differentiated CTLs (Supplementary Fig. 3h, i). Consistent with impaired mitochondrial respiration in mPiC-deficient T cells, we observed lower glucose-derived ^13^C-labeling of TCA cycle intermediates, whereas the fractional enrichment of ^13^C in glycolytic metabolites and lactate was unaltered or increased (Supplementary Fig. 3j, k). Cellular ATP levels were unaltered in mPiC-deficient T cells upon antigen receptor stimulation (Fig. 2c), suggesting that elevated aerobic glycolysis compensates for the impaired mitochondrial ATP biosynthesis. This notion is in line with a higher ECAR:OCR ratio (Supplementary Fig. 3i) and a greater bioenergetic dependency on aerobic glycolysis of mPiC-deficient T cells (Fig. 2d). The observation that both Tex (ex vivo) and mPiC-deficient T cells (in vitro) prefer aerobic glycolysis over mitochondrial respiration highlights the potential of *Slc25a3*^fl/fl^*Cd4*^Cre^ mice to explore the mechanistic link between metabolic reprogramming and T cell exhaustion.

To further investigate this hypothesis, we performed bulk RNA sequencing to delineate how metabolic reprogramming affects gene expression of activated and CTL-differentiated WT and mPiC-deficient T cells (Supplementary Fig. 3l, m). The gene expression profiles of both genotypes were clearly distinct and revealed 1475 and 463 differentially expressed genes (DEGs) in activated T cells and CTLs, respectively (Supplementary Fig. 3l, m). To explore the physiological processes governed by mPiC-dependent metabolic reprogramming, we performed pathway enrichment and network cluster analyzes (Fig. 2e). Surprisingly, gene signatures correlating with *immune cell activation* and *lymphocyte differentiation* were enriched in mPiC-deficient T cells after activation (Fig. 2e), suggesting that T cell activation is not impaired, but rather enhanced, when mitochondrial ATP synthesis is substituted by aerobic glycolysis. Indeed, gene expression signatures involved in *mitochondrial respiration*, *mitochondrial translation*, and *oxidative phosphorylation* were lower in mPiC-deficient T cells (Fig. 2e, f). The gene expression profiles of mPiC-deficient T cells were poorly correlated with those of effector T cells but were shifted towards an exhaustion signature (Fig. 2g). In fact, numerous genes that are characteristic for T cell exhaustion, such as *Havcr2*, *Pdcd1*, *Lag3* and *Gzma*, were markedly upregulated in mPiC-deficient CTLs (day 6), whereas Tpex-like genes, including *Sell*, *Ccr7*, *Tcf7* and *Slamf6*, were downregulated (Fig. 2h). Consistent with the transcriptomic findings, Tim-3, PD-1, Lag3 and granzyme A protein levels were also higher in mPiC-deficient CTLs compared to WT controls (Fig. 2i, j). Intriguingly, when we differentiated WT and mPiC-deficient T cells with IL-7 and IL-15 to generate ‘memory-like’ T cells in vitro, we did not observe an elevated expression of co-inhibitory receptors (Supplementary Fig. 4a–c). However, CD62L and Ly108 expression were impaired in mPiC-deficient memory-like T cells (Supplementary Fig. 4b), indicating that mitochondrial respiration supports the maintenance of both Tpex and memory-like T cells. In addition to the ‘acute’ activation of T cells with anti-CD3/CD28 (Fig. 2i, j and Supplementary Fig. 4a–c), we also stimulated WT and mPiC-deficient T cells ‘chronically’ to provoke exhaustion by continuous antigenic stimulation^21^ (Supplementary Fig. 4d–g). As expected, chronic stimulation massively upregulated the expression of activation markers and co-inhibitory receptors (Supplementary Fig. 4d–f). Although many of these markers were already maximally expressed under these conditions, Tim-3 expression was significantly higher in mPiC-deficient T cells compared to WT controls (Supplementary Fig. 4f). To demonstrate that mPiC-deficient T cells are also functionally exhausted, we analyzed the cytokine production profile (Fig. 2l and Supplementary Fig. 2g) and apoptosis (Fig. 2m). As expected, mPiC-deficient T cells produced significantly less IFNγ and IL-2 under ‘acute’ and ‘chronic’ culture conditions (Fig. 2l and Supplementary Fig. 4g) and showed impaired survival after antigen receptor ligation (Fig. 2m). Collectively, these data demonstrate that mPiC ablation causes both phenotypic and functional exhaustion of T cells.

**Fig. 3:**
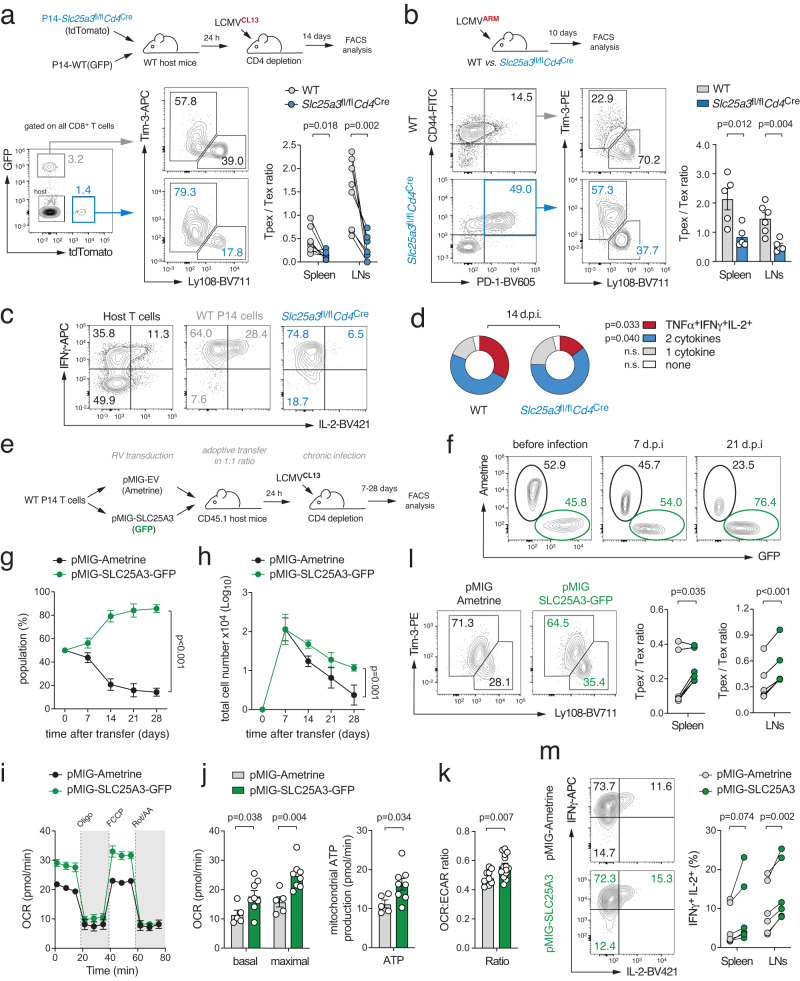
Mitochondrial respiration controls the functional exhaustion of virus-specific T cells. **a** Adoptive co-transfer of GFP^+^ WT and tdTomato^+^ mPiC-deficient (*Slc25a3*^fl/fl^*Cd4*^Cre^) P14 T cells into C57BL/6 mice before chronic infection with LCMV clone 13 (LCMV^CL13^). Flow cytometric analysis of Tpex and Tex cells in spleen and LNs of the host mice 14 days post infection (d.p.i.); *n* = 8 mice. **b** Acute infection of WT and *Slc25a3*^fl/fl^*Cd4*^Cre^ mice with LCMV Armstrong (LCMV^ARM^). Analysis of Tpex and Tex cells was performed 10 d.p.i.; means ± SEM of 5 mice. **c**, **d** Analysis of TNFα, IFNγ, and IL-2 expression after PMA/iono restimulation of WT and mPiC-deficient P14 T cells after co-transfer into chronically infected mice; means ± SEM of 6–10 mice. **e**–**m** Ectopic expression of mPiC attenuates T cell exhaustion. **e** Retroviral transduction of WT P14 T cells with GFP^+^ SLC25A3/mPiC or Ametrine^+^ empty vector followed by adoptive co-transfer into chronically infected mice. **f** Representative flow cytometric analysis of GFP^+^ and Ametrine^+^ P14 T cells after transfer into LCMV^CL13^ infected CD45.1^+^ mice. **g**, **h** Relative and absolute numbers of GFP^+^ and Ametrine^+^ P14 T cells in the spleens; means ± SEM of 2-6 mice. **i**, **j** Basal and maximal oxygen consumption rate (OCR) (**i**) and mitochondrial ATP production rate (**j**) in mPiC (GFP^+^) and empty vector (Ametrine^+^) transduced P14 cells ex vivo 7 d.p.i. using a Seahorse extracellular flux analyzer; means ± SEM of 3 mice analyzed in 2-3 technical replicates. **k** Ratio of OCR to extracellular acidification rate (ECAR) in mPiC overexpressing *versus* empty vector T cells; means ± SEM of 3 mice analyzed in 5 technical replicates. **l** Flow cytometric analysis of Tpex and Tex cell ratio in mPiC (GFP^+^) and empty vector transduced (Ametrine^+^) P14 cells 7 d.p.i.; *n* = 6 mice. **m** IFNγ and IL-2 expression in mPiC and empty vector transduced P14 cells after restimulation 7 d.p.i.; means ± SEM of 6 mice. Paired and two-tailed unpaired Student’s t-test in (**a**), (**b**), (**d**), and (**i**–**m**) or 2-way ANOVA in (**g**, **h**).

Based on the observation that mitochondrial (dys-)function affects the expression of both stemness and exhaustion markers in vitro (Fig. 2h–k), we next investigated how mitochondrial insufficiency affects T cell differentiation during chronic viral infection in vivo. To track antigen-specific T cells, we crossed WT and *Slc25a3*^fl/fl^*Cd4*^Cre^ mice to P14 animals that express an LCMV-specific T cell receptor and fluorescent reporters. 14 days after adoptive co-transfer of WT (GFP^+^) and mPiC-deficient P14 T cells (tdTomato^+^) into chronically infected WT host mice, we analyzed the proportions of Tpex and Tex cells among the donor T cell populations (Fig. 3a). The frequencies of Tpex cells were ~ 50% reduced in mPiC-deficient T cells compared to those of WT T cells, whereas the proportion of Tex cells was reciprocally increased in absence of mPiC (Fig. 3a and Supplementary Fig. 5a). Of note, GFP^+^ WT T cells numerically outcompeted mPiC-deficient P14 cells over time (Supplementary Fig. 5b, c). The loss of mPiC-deficient T cells may be explained by an impaired clonal expansion, elevated apoptosis or both. Indeed, mPiC-deficient T cells showed both impaired proliferation (Supplementary Fig. 5d) and increased apoptosis (Fig. 2m), which also affected their clonal expansion in vitro (Supplementary Fig. 5e). Adoptive co-transfer experiments of cell trace violet (CTV)-labeled T cells confirmed a reduced proliferative capacity of mPiC-deficient T cells in vivo (Supplementary Fig. 5f), providing a plausible explanation for their competitive disadvantage. These findings suggest that mitochondrial respiration is required for the maintenance of Tpex cells during chronic infection and/or that mitochondrial insufficiency accelerates terminal T cell exhaustion. To test the latter hypothesis, we infected WT and *Slc25a3*^fl/fl^*Cd4*^Cre^ mice with the acute ‘Armstrong’ strain of LCMV (LCMV^ARM^), which provokes a transient infection in WT mice without causing T cell exhaustion. T cells of LCMV^ARM^ infected WT mice displayed the expected effector phenotype and the few PD-1^+^ T cells co-expressed Ly108 (Fig. 3b). By contrast, the frequency of PD-1^+^ T cells was dramatically increased in *Slc25a3*^fl/fl^*Cd4*^Cre^ mice, of which ~ 60% were positive for Tim-3 (Fig. 3b). Furthermore, mPiC-deficient T cells showed a markedly reduced capability to produce cytokines after restimulation (Fig. 3c, d), demonstrating that defective mitochondrial respiration not only causes phenotypic features of T cell exhaustion but also results in dysfunction. Conversely, to test the hypothesis that augmented mitochondrial respiration sustains the stemness of exhausted T cells, we retrovirally (RV) overexpressed mPiC in naïve T cells and adoptively co-transferred control (pMIG-Ametrine^+^) and mPIC-overexpressing P14 T cells (pMIG-SLC25A3-GFP^+^) into LCMV^CL13^ infected recipient mice (Fig. 3e). Strikingly, mPiC-overexpressing T cells outcompeted control cells over time (Fig. 3f–h), suggesting that augmented mitochondrial respiration (Fig. 3i–k) protects virus-specific T cells from functional exhaustion. Indeed, phenotypic and functional analyzes of donor T cells revealed that mPiC-overexpressing T cells retained a higher proportion of Tpex cells (Fig. 3l) and a superior capacity to produce cytokines (Fig. 3m).

Taken together, these findings show that defects in mitochondrial respiration are sufficient to elicit the transcriptional, phenotypic and functional features of T cell exhaustion even in the absence of continuous antigen exposure.

### Oxidative stress stabilizes HIF-1α protein levels in Tpex cells

**Fig. 4:**
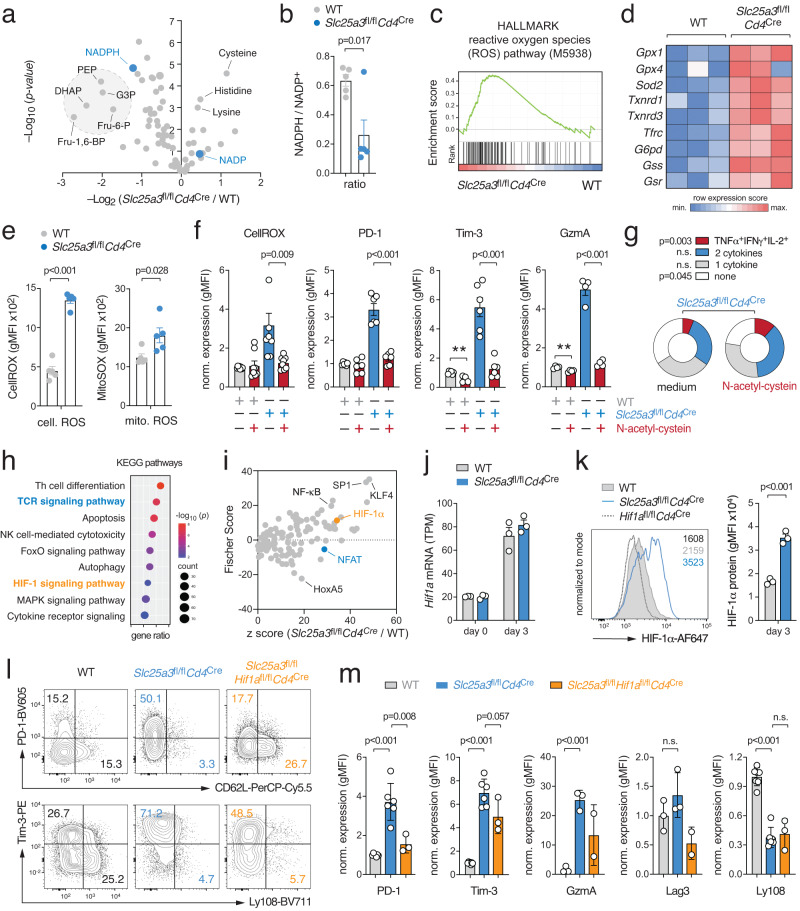
Mitochondrial insufficiency causes ROS-mediated HIF-1 α protein stabilization. **a** Volcano plot of differential metabolite concentrations of in vitro activated WT and mPiC-deficient (*Slc25a3*^fl/fl^*Cd4*^Cre^) T cells analyzed by untargeted liquid chromatography and mass spectrometry (LC/MS); means ± SEM of 4 mice. **b** Analysis of NADPH/NADP^+^ ratios in WT and mPiC-deficient T cells using LC/MS; means ± SEM of 4 mice. **c** Gene set enrichment analysis (GSEA) of the *reactive oxygen species (ROS) pathway* gene signature in WT and mPiC-deficient T cells. **d** Heatmap analysis of selected genes involved in ROS detoxification. **e** Flow cytometric analysis of cellular (CellROX) and mitochondrial ROS (MitoSox) in WT and mPiC-deficient T cells; means ± SEM of 5 mice. **f** Scavenging of ROS by N-acetylcysteine (NAC) prevents exhaustion of mPiC-deficient T cells. Flow cytometric analyzes of CellRox, PD-1, Tim-3 and granzyme A expression in WT and mPiC-deficient T cells treated with NAC; means ± SEM of 4–8 mice. **g** Analysis of TNFα, IFNγ and IL-2 expression after PMA/iono restimulation of mPiC-deficient T cells treated with or without NAC; means ± SEM of 5 mice. **h** Kyoto Encyclopedia of Genes and Genomes (KEGG) pathway enrichment analysis using bulk RNA sequencing data of WT and mPiC-deficient T cells. **i** Upstream transcription factor prediction analysis using differentially expressed genes (DEGs) between WT and mPiC-deficient T cells. **j** Analysis of *Hif1a* gene expression in WT and mPiC-deficient T cells by RNA sequencing; means ± SEM of 3 mice. **k** Flow cytometric analysis of HIF-1α protein expression in WT, mPiC- and HIF-1α-deficient T cells; means ± SEM of 3 mice. **l**, **m** Differentiation of WT, mPiC-deficient (*Slc25a3*^fl/fl^*Cd4*^Cre^) and mPiC/HIF-1α double-deficient (*Slc25a3*^fl/fl^*Hif1a*^fl/fl^*Cd4*^Cre^) T cells in vitro. Representative flow cytometric analysis (**l**) and quantification of PD-1, Tim-3, granzyme A, Lag3 and Ly108 expression on WT, mPiC-deficient and mPiC/HIF-1α double-deficient T cells (**m**); means ± SEM of 2–7 mice. Two-tailed unpaired Student’s *t*-test in (**b**), (**e–g**), (**k**) and (**m**).

To understand how the metabolic changes in mPiC-deficient T cells control the complex transcriptional processes during T cell exhaustion, we profiled their metabolome using liquid chromatography and mass spectrometry (LC/MS) (Fig. 4a). Among the most downregulated metabolites in mPiC-deficient T cells were several glycolytic intermediates, such as *fructose-6-phosphate*, *fructose-1,6-bisphospate*, *dihydroxyacetone-phosphate* (DHAP), *glyceraldehyde-3-phosphate* (G3P) and *phosphoenolpyruvate* (PEP), supposably due to an accelerated glycolytic flux. In addition, the reduced form of *nicotinamide adenine dinucleotide phosphate* (NADPH) was downregulated in mPiC-deficient T cells (Fig. 4a), resulting in a shifted NADPH/NADP^+^ balance (Fig. 4b). The regeneration of cytosolic NADPH is linked to the pentose phosphate pathway (PPP) and metabolically coupled to the conversion of glucose-6-phosphate (G6P) to pentose-5-phosphate^30^. We next used stable-isotope tracing to determine the glucose-derived ^13^C-labeling pattern of TCA cycle and PPP intermediates. Intriguingly, ^13^C-pentose-5-phosphate was markedly reduced in mPiC-deficient T cells (Supplementary Fig. 6a) and the gene expression signature of the PPP declined progressively during T cell exhaustion (Supplementary Fig. 6b). These findings indicate that glucose is re-directed into glycolysis to compensate for the impaired mitochondrial ATP production rate, thereby limiting NADPH production. Given the crucial role of NADPH for the cellular redox hemostasis as an antioxidant^30^, we hypothesized that oxidative stress promotes the exhaustion phenotype of mPiC-deficient T cells. This notion was supported by GSEA which revealed a strong correlation with the *reactive oxygen species (ROS) pathway* gene expression signature (M5938) (Fig. 4c) and an upregulation of genes involved in ROS detoxification (Fig. 4d). Flow cytometric analyzes using CellROX and MitoSox revealed elevated cytosolic and mitochondrial ROS levels, respectively (Fig. 4e). To test whether neutralization of cellular ROS can rescue mPiC-deficient T cells from functional exhaustion, we supplemented WT and mPiC-deficient T cells with N-acetylcysteine (NAC). In line with recent reports^20,21^, NAC normalized cellular ROS levels in mPiC-deficient T cells, prevented the expression of exhaustion markers (Fig. 4f), and restored their cytokine production (Fig. 4g).

We next addressed how elevated ROS levels are translated into gene expression in mPiC-deficient T cells. To identify potential signaling pathways and transcriptional regulators, we performed pathway (Fig. 4h) and transcription factor enrichment analyzes (Fig. 4i). Both analyzes highlighted an involvement of TCR and NFAT signaling (Fig. 4h, i). This was intriguing because NFAT controls numerous genes involved in T cell exhaustion^16,31–33^. It has been shown that mitochondrial-derived ROS can sustain the nuclear translocation of NFAT during T cell activation^20,21,34,35^ (Supplementary Fig. 6c). Chronically stimulated T cells express predominantly the short isoform of NFATc1^36,16,37^. To test whether NFATc1 also drives exhaustion of mPiC-deficient CTLs, we crossed *Nfatc1*^fl/fl^ to *Slc25a3*^fl/fl^*Cd4*^Cre^ mice to obtain mPiC/NFATc1 double-deficient T cells. Surprisingly, ablation of NFATc1 did not prevent the upregulation of PD-1, Tim-3 and other co-inhibitory receptors in mPiC-deficient T cells (Supplementary Fig. 6d, e), suggesting that different NFAT family members can compensate for the loss of NFATc1 and/or that other transcription factors promote exhaustion of mPiC-deficient T cells. Supporting the latter, pathway and transcription factor enrichment analyzes identified HIF-1α as a potential regulator (Fig. 4h, i) and gene and protein levels of HIF-1α were markedly increased in Tpex and transitory T cells (Supplementary Fig. 6f, g). Furthermore, HIF-1α target gene expression peaked during the transition of Tpex into Tex cells (Supplementary Fig. 6h), indicating that HIF-1α signaling contributes to T cell exhaustion. The activity of HIF-1α is regulated at the transcriptional and posttranslational level^38,39^. The protein stability of HIF-1α is dependent on cellular oxygen tension because prolyl hydoxylases (PHDs) catalyze the hydroxylation of HIF-1α in normoxia, which promotes its ubiquitination and proteasomal degradation. Under hypoxia – or when the enzymatic activity of PHD is inhibited – HIF-1α protein is stabilized and dimerizes with ARNT to form an active transcription factor complex^38,39^. Importantly, PHDs require α-ketoglutarate, Fe^2+^ and ascorbate as cofactors and ROS-mediated oxidation of these cofactors inhibits the enzymatic activity of PHD (Supplementary Fig. 6i)^39,40^. While gene expression of *Hif1a* was comparable between WT and mPiC-deficient T cells in vitro (Fig. 4j), HIF-1α protein levels were dramatically increased in mPiC-deficient T cells (Fig. 4k). The hydroxylation of HIF-1α is metabolically coupled to the conversion of α-ketoglutarate to succinate, which was reduced in mPiC-deficient T cells (Supplementary Fig. 6j). Importantly, scavenging ROS by NAC normalized HIF-1α protein expression levels in mPiC-deficient T cells (Supplementary Fig. 6k), demonstrating that ROS prevent the proteasomal degradation of HIF-1α protein. To examine whether HIF-1α contributes to the upregulation of co-inhibitory receptors in mPiC-deficient T cells, we crossed *Hif1a*^fl/fl^ to *Slc25a3*^fl/fl^*Cd4*^Cre^ mice to generate mPiC/HIF-1α double-deficient T cells. Ablation of HIF-1α attenuated the expression of PD-1, Tim-3, and Lag3 in mPiC-deficient T cells, but failed to restore the expression of Ly108 (Fig. 4l, m).

Parallel to mPiC-deficient mice, we established an additional genetic model of mitochondrial dysfunction by generating mice with T cell-specific ablation of TFAM (*Transcription factor A, mitochondrial*) (Supplementary Fig. 7). TFAM was highly expressed in Tpex cells but almost completely lost in terminally exhausted T cells (Supplementary Fig. 2d). Because TFAM plays a crucial role in regulating mitochondrial DNA transcription and replication, its ablation efficiently inhibits mitochondrial biogenesis and oxidative phosphorylation^41,42^. Similar as observed for mPiC ablation, TFAM-deficient T cells also showed the characteristic metabolic switch from mitochondrial respiration to aerobic glycolysis (Supplementary Fig. 7a, b), accumulation of cellular ROS (Supplementary Fig. 7c) and the posttranslational stabilization of HIF-1α (Supplementary Fig. 7d), which was correlated with an upregulation of different exhaustion markers (Supplementary Fig. 7e, f).

Collectively, these data demonstrate that oxidative stress and metabolic reprogramming due to mitochondrial insufficiency supports HIF-1α protein stabilization and T cell exhaustion.

### HIF-1α-mediated glycolytic reprogramming supports terminal T cell differentiation

**Fig. 5:**
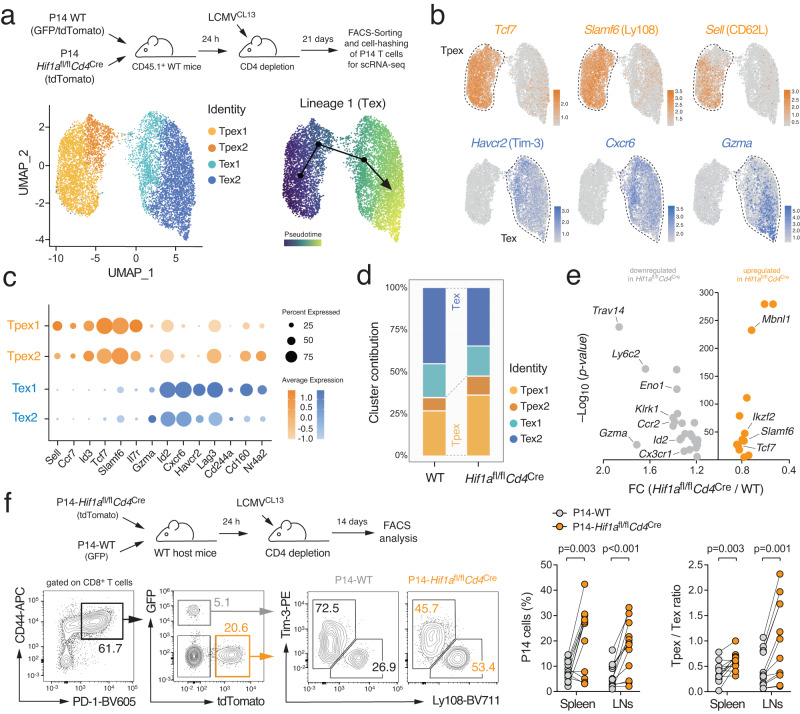
HIF-1α controls terminal differentiation of virus-specific T cells. **a** Adoptive co-transfer of WT (tdTomato^+^GFP^+^) and HIF-1α-deficient (tdTomato^+^) P14 T cells into C57BL/6 mice before chronic infection with LCMV clone 13 (LCMV^CL13^). 21 days post infection (d.p.i.), donor P14 WT and HIF-1α-deficient T cells were FACS sorted, barcoded, and multiplexed in a 1:1 ratio and subjected to single-cell RNA sequencing. Uniform manifold approximation and projection (UMAP) visualization of ~8.400 non-proliferating (*Mki67* low/negative) T cells identified four clusters. Prediction of developmental trajectories using slingshot analysis; color-coding according to pseudotime. **b** Normalized gene expression of *Tcf7*, *Slamf6* (Ly108), *Sell* (CD62L), *Havcr2* (Tim-3), *Cxcr6* and *Gzma* projected onto UMAP clusters. **c** Dot plot analysis of selected cluster markers representing Tpex and Tex cell subsets; color intensity and dot size represent z-score mean expression and percentage of cells expressing the gene, respectively. **d** Relative contribution of individual UMAP clusters in WT and Hif-1α-deficient P14 T cells. **e** Volcano plot of differentially expressed genes (DEGs) between WT and HIF-1α-deficient P14 T cells using scRNA sequencing. **f** Adoptive co-transfer of WT (GFP^+^) and HIF-1α-deficient (tdTomato^+^) P14 T cells into C57BL/6 mice before chronic infection with LCMV^CL13^. Flow cytometric analysis of Tpex and Tex cells was performed 14 d.p.i.; *n* = 12 mice. Two-tailed paired Student’s t-test in (**f**).

We next investigated how HIF-1α ablation affects terminal T-cell exhaustion during chronic viral infection. We crossed *Hif1a*^fl/fl^*Cd4*^Cre^ mice to P14 animals and adoptively co-transferred equal numbers of WT (GFP^+^) and HIF-1α-deficient T cells (tdTomato^+^) into chronically infected WT host mice. 21 days post infection, we retrieved GFP^+^ and tdTomato^+^ donor T cells and subjected individually barcoded samples to scRNA sequencing analysis (Supplementary Fig. 8a). Using a similar analysis strategy as for polyclonal T cells (Fig. 1a–e), we identified several UMAP clusters representing precursor and terminally exhausted T cells (Fig. 5a, b and Supplementary Fig. 8b, c). Both precursor populations expressed the ‘stemness’ markers *Tcf7* and *Slamf6*, but Tpex2 differed from Tpex1 cells by the loss of *Sell* and *Il7r* (Fig. 5b, c and Supplementary Fig. 8b, c). The two terminally exhausted T cell populations were characterized by the expression of *Havcr2*, *Cxcr6*, *Id2*, *Lag3*, *Gzma* and *Cd244a* (Fig. 5b, c and Supplementary Fig. 8b, c). Diffusion pseudotime analysis predicted a unidirectional trajectory originating from stem-like CD62L^+^ Tpex1 cells^9^ into terminally differentiated Tex cells (Fig. 5a). Similar as polyclonal T cells (Fig. 1e), P14 T cells also showed a sharp decline in their mitochondrial gene signatures when progressing from Tpex to Tex cells (Supplementary Fig. 8d). Importantly, HIF-1α-deficient T cells were enriched within the Tpex populations, whereas most WT T cells were found in terminally exhausted UMAP clusters (Fig. 5d). Comparing the DEGs between WT and HIF-1α-deficient P14 T cells across clusters, we found that *Tcf7* and *Slamf6* were significantly higher in HIF-1α-deficient T cells, whereas exhaustion-associated genes were markedly decreased (Fig. 5e). To test our hypothesis that HIF-1α supports the transition of Tpex into terminally exhausted T cells, we adoptively co-transferred WT and HIF-1α-deficient P14 T cells into chronically infected WT host mice. 14 days after transfer, we found a significantly increased Tpex cell population, but fewer terminally exhausted T cells, among HIF-1α-deficient donor T cells (Fig. 5f). Similarly, when we analyzed endogenous T cell responses in WT and *Hif1a*^fl/fl^*Cd4*^Cre^ mice after chronic LCMV infection, we also observed a higher Tpex-to-Tex ratio among the virus-specific T cells in HIF-1α-deficient mice (Supplementary Fig. 8e, f).

**Fig. 6:**
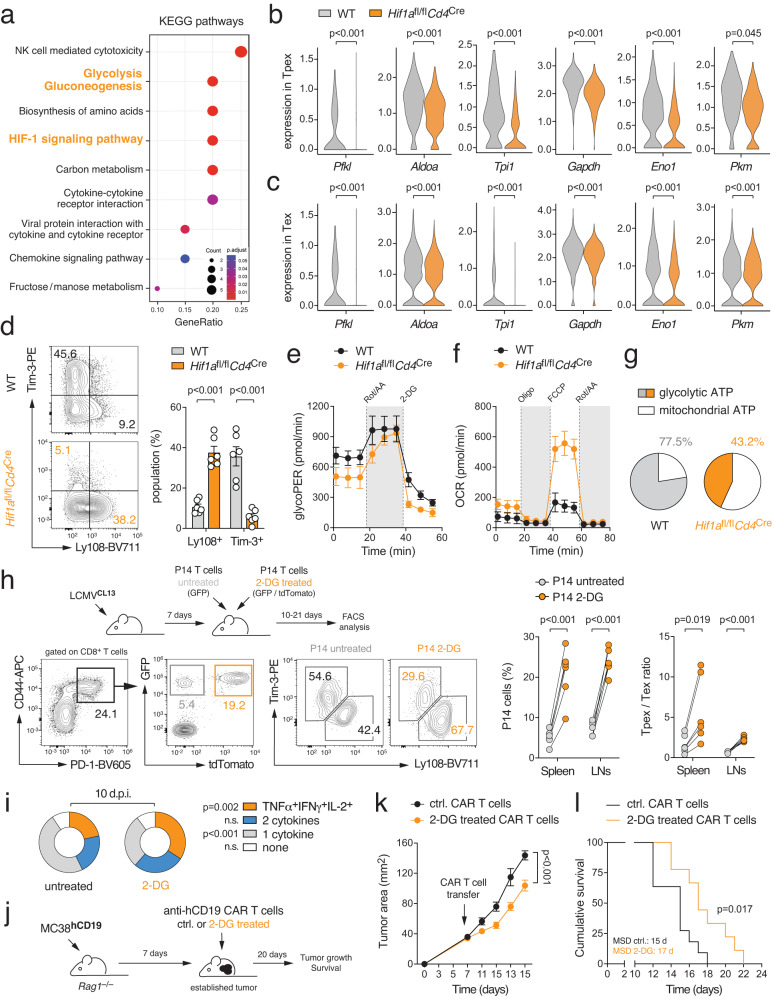
HIF-1α-mediated glycolytic reprogramming promotes T cell exhaustion. **a** KEGG pathway enrichment analysis using single-cell RNA sequencing of WT and HIF-1α-deficient T cells. **b**, **c** Violin plots displaying *Pfkl, Aldoa, Tpi1, Gapdh*, *Eno1,* and *Pkm* gene expression in Tpex (**b**) and Tex cells (**c**) of WT and HIF-1α-deficient (*Hif1a*^fl/fl^*Cd4*^Cre^) P14 T cells analyzed by single-cell RNA sequencing. **d** Differentiation of WT and HIF-1α-deficient T cells under hypoxia in vitro, means ± SEM of 6 mice. **e**, **f** Analyzes of glycolytic proton efflux rate (glycoPER) (**e**) and oxygen consumption rate (OCR) (**f**) in WT and HIF-1α-deficient T cells using a Seahorse extracellular flux analyzer; means ± SEM of 3 mice. **g** Relative contribution of glycolysis and mitochondrial respiration to cellular ATP production in WT and HIF-1α-deficient T cells; means ± SEM of 3 mice. **h**, **i** Inhibition of glycolysis sustains the stemness of virus-specific T cells. **h** Adoptive co-transfer of 2-DG treated (tdTomato^+^ GFP^+^) and control (GFP^+^) P14 T cells into C57BL/6 mice after chronic infection with LCMV^CL13^. Flow cytometric analysis of Tpex and Tex cells among donor P14 cells 10 d.p.i.; *n* = 6 mice. **i** Analysis of TNFα, IFNγ, and IL-2 expression by 2-DG-treated and control P14 T cells 10 days after co-transfer into chronically infected mice; means ± SEM of 6 mice. **j**–**l** 2-DG treatment augments the antitumor efficacy of CAR T cells. **j** A total of 1 × 10^6^ anti-hCD19 CAR T cells treated with or without 2-DG for 24 h were adoptively transferred into *Rag1*^–/–^ mice 7 days after MC38 tumor inoculation. **k**, **l** Analysis of tumor growth (**k**) and cumulative survival (**l**) of tumor-bearing host mice after transfer of 2-DG or control-treated CAR T cells; means ± SEM of 9–11 mice. Unpaired and two-tailed paired Student’s t-test in (**b**–**d**) and (**h**). In (**k**) and (**l**), 2-way ANOVA and Mantel-Cox test, respectively.

To decipher how HIF-1α supports the exhaustion of virus-specific T cells, we performed pathway enrichment analyzes based on the DEGs between WT and HIF-1α-deficient P14 T cells (Fig. 5a–e). Besides the expected association with the *HIF-1α signaling pathway*, we found a strong correlation of the DEGs with the *glycolysis and gluconeogenesis* gene signature (Fig. 6a). The expression of glycolytic enzymes, such as *phosphofructokinase* (*Pfkl*), *aldolase* (*Aldoa*), *glyceraldehyde-3-phosphate dehydrogenase* (*Gapdh*), *enolase* (*Eno1*) and *pyruvate kinase* (*Pkm*), were significantly downregulated in HIF-1α-deficient Tpex and Tex cells (Fig. 6b, c). Accordingly, HIF-1α-deficient T cells showed reduced glycolytic activity in vitro, which was associated with a lower expression of co-inhibitory receptors (Fig. 6d, e and Supplementary Fig. 9a). Intriguingly, however, HIF-1α-deficient CTLs compensated their glycolytic deficit by elevated OXPHOS (Fig. 6f). The higher bioenergetic dependency on mitochondrial respiration (Fig. 6g) correlated with an increased CD62L and Ly108 expression (Supplementary Fig. 9b), suggesting that HIF-1α-mediated glycolytic reprogramming promotes the differentiation of Tpex into Tex cells. Indeed, 2-deoxy-D-glucose (2-DG), a potent inhibitor of glycolysis, prevented the upregulation of Tim-3, Lag-3 and granzyme A expression in mPiC-deficient T cells (Supplementary Fig. 9c). To test whether glycolytic restriction also preserves the stemness of virus-specific T cells in vivo, we stimulated P14 T cells with anti-CD3/CD28 in vitro followed by 2-DG treatment for 24 h before we co-transferred equal numbers of 2-DG-treated (tdTomato^+^GFP^+^) and control (GFP^+^) T cells into LCMV infected host mice (Fig. 6h and Supplementary Fig. 9d–f). 2-DG-treated P14 cells outcompeted control T cells 10 and 21 days after transfer (Fig. 6h and Supplementary Fig. 9f) and retained more Tpex cells at both timepoints (Fig. 6h and Supplementary Fig. 9d, e). Of note, 2-DG-treated T cells were fully functional after transfer into chronically infected mice (Fig. 6i and Supplementary Fig. 9g), demonstrating that short-term inhibition of glycolysis preserves the stemness of virus-specific T cells without compromising their effector functions. To further address whether 2-DG also prevents terminal exhaustion of T cells in the context of antitumor immunity, we stimulated ovalbumin (OVA)-specific OT-1 T cells with anti-CD3/CD28 for 2 days in vitro followed by 2-DG treatment for 24 h before transfer into mice bearing MC38 colon carcinomas expressing OVA (Supplementary Fig. 9h–l). Short term-treatment of OT-1 T cells with 2-DG increased the OCR:ECAR ratio (Supplementary Fig. 9i) and their bioenergetic dependency on mitochondrial respiration (Supplementary Fig. 9j), which correlated with lower expression of co-inhibitory receptors (Supplementary Fig. 9k) and greater antitumor immunity (Supplementary Fig. 9l). These findings provide a rationale for the generation of ‘exhaustion-resistant’ CAR T cells for cancer immunotherapy. To test this concept, we transduced naive T cells with a second-generation CAR targeting human CD19^33^. After retroviral transduction with the CAR construct, activated T cells were treated with 2-DG for 24 h before a suboptimal number of 1×10^6^ CAR T cells was adoptively transferred into lymphopenic host mice bearing hCD19-expressing MC38 tumors (Fig. 6j). Similar as OVA-specific CTLs, 2-DG pretreated CAR T cells also showed increased antitumor activity, resulting in improved tumor regression (Fig. 6k) and prolonged survival (Fig. 6l).

All together, these findings suggest that both mitochondrial insufficiency and HIF-1α-mediated glycolytic reprogramming contribute to T cell exhaustion and that pharmacological inhibition of glycolytic reprogramming is a feasible metabolic intervention strategy to maintain the stemness, longevity, and functionality of (CAR) T cells during chronic viral infection and cancer immunotherapy.

## Discussion

Exhausted T cells are heterogenous and comprise developmentally and functionally distinct subsets that differ in their transcriptional, epigenetic, and metabolic signatures^9,20–24,26,43^. By analyzing single-cell gene expression profiles of exhausted T cell subsets, we found that the transition of stem-like Tpex into functionally exhausted T cells is paralleled by a sharp decline in mitochondrial gene expression and metabolic reprogramming towards aerobic glycolysis. Using a novel genetic mouse model of mitochondrial insufficiency in T cells, we here show that this metabolic switch is sufficient to induce the exhaustion-associated gene expression program in Tpex cells, thus resembling an important cell-intrinsic trigger and not merely the consequence of exhaustion. Mitochondrial decline and the re-direction of glucose into aerobic glycolysis diminishes the generation of NADPH, causing redox stress and an accumulation of ROS. Pathway and transcription factor enrichment analyzes identified HIF-1α as an important transcriptional regulator promoting glycolytic reprogramming and terminal T-cell exhaustion in response to mitochondrial insufficiency.

Mitochondria play a pivotal role in cellular metabolism by generating energy in the form of ATP. The respiratory chain complexes generate a proton gradient following OXPHOS at the inner mitochondrial membrane that is utilized by the ATP synthase to generate ATP from ADP and inorganic phosphate^13,14,39^. In addition to their bioenergetic function, mitochondria provide intermediary metabolites controlling the fate and function of T cells by regulating signaling pathways, redox balance, apoptosis, and epigenetic rewiring through post-translational DNA and histone modifications^28,39,44^. Recent studies revealed that mitochondrial insufficiency, characterized by membrane depolarization, metabolic alterations, and the production of ROS, correlates with the functional exhaustion of T cells^20–24^. Yet, a key challenge in cellular metabolism is to delineate if these changes are the cause or a consequence of T cell exhaustion. By combining novel genetic models of mitochondrial insufficiency with single-cell and functional analyzes, we here show that metabolic remodeling is sufficient to elicit the exhaustion-associated gene expression program and dysfunction in Tpex cells. Mitochondrial dysfunction provoked T cell exhaustion even in the absence of continued antigen exposure, suggesting that a decline in mitochondrial respiration acts – in analogy to Knudson’s “two-hit” theory in cancer^45^ – as a second hit to initiate the exhaustion-associated gene expression program in T cells.

To establish direct causality between mitochondrial insufficiency and T cell exhaustion, we ablated the *mitochondrial phosphate carrier* (mPiC), encoded by the *Slc25a3* gene^29,46^. Although several studies have linked altered mitochondrial metabolism to T cell exhaustion in vitro^20,21^, during chronic viral infection^22,23^ and in cancer^24,25^, we here provide genetic evidence that impaired mitochondrial respiration is not merely a consequence of T cell dysfunction but, instead, promotes the transcriptional, phenotypic and functional hallmarks of exhaustion. Supporting the notion that mitochondrial dysfunction acts as a “second hit” for terminal T cell differentiation, mPiC-deficient Tpex cells did not only show an accelerated differentiation into terminally exhausted T cells during chronic viral infection, but also showed an exhaustion-associated gene expression signature under circumstances that are not expected to cause exhaustion, such as in acute stimulation.

A simple explanation for the exhaustion in response to mitochondrial deterioration in mPiC-deficient T cells could be a paucity in ATP, which was, however, not the case. mPiC-deficient T cells compensated their mitochondrial deficit by a ‘Warburg effect-like’ metabolic switch to aerobic glycolysis, thus preventing a fatal bioenergetic crisis. However, when glucose is primarily converted into lactate to compensate for the defective mitochondrial ATP production, intermediary metabolites become limiting in biochemical reactions that generate reduction equivalents, such as NADPH in the oxidative branch of the *pentose phosphate pathway* (PPP)^14,30^. Given the crucial role of NADPH as a cellular antioxidant^30,39^, our results suggest that ROS is not solely a by-product of defective mitochondrial metabolism, but a molecular rheostat that links mitochondrial performance to T cell dysfunction^20,21,24^.

A recent report by Scharping et al. demonstrated that hypoxia and continuous antigenic stimulation promote T cell exhaustion through Blimp-mediated repression of PCG-1α and mitochondrial dysfunction^20^. In this study, hypoxia-induced mitochondrial ROS elicited the exhaustion-associated gene expression via NFAT signaling^20^. In our model, T cell exhaustion occurred even in normoxia, suggesting that defective mitochondrial respiration is a cell-autonomous trigger for T cell exhaustion, independent of hypoxic exposure and other exogenous metabolic stressors. Although NFAT’s activity can be fine-tuned by cellular ROS and promotes T cell exhaustion^16,32,33,36,37^, ablation of NFATc1 did not halt terminal differentiation of mPiC-deficient T cells. Using pathway and transcription factor enrichment analyzes, we identified HIF-1α as an alternative, redox-regulated regulator of T cell dysfunction. Supporting our hypothesis that HIF-1α supports terminal T cell exhaustion in response to excessive ROS production, ablation of HIF-1α alleviated terminal differentiation of both genetically and virally exhausted T cells. Our findings are consistent with previous studies that revealed reduced expression of exhaustion-associated proteins in HIF-1α-deficient T cells^47–49^, whereas stabilization of HIF-1α through genetic inactivation of its negative regulators increased the expression of co-inhibitory receptors^50–53^. These data suggest that HIF-1α contributes – at least in part – to T cell dysfunction during chronic antigenic stimulation by promoting the transition of Tpex into terminally exhausted T cells.

It is important to mention that constitutive HIF-1α signaling also maintains the effector function of T cells during chronic viral infection and cancer. T cells with enhanced HIF-1α activity sustain expression of effector molecules, are refractory to exhaustion, and demonstrate superior control of persistent infection and tumors^50,52,54^. Thus, HIF-1α appears to play a Janus-faced role in chronically stimulated T cells by supporting their inflammatory function on the one hand and accelerating their exhaustion on the other. This is reminiscent of NFAT transcription factors, which are critical regulators of both T cell function and exhaustion^16^. It is tempting to speculate that a similar scenario also applies to HIF-1α. While constitutive HIF-1α signaling or hypoxia promotes the glycolytic reprogramming and effector differentiation of CTLs, HIF-1α may also accelerate T cell dysfunction in response to mitochondrial insufficiency and intracellular ROS. However, these opposing effects of HIF-1α may not occur in the same cells since exhausted T cells are a heterogenous population. It is conceivable that constitutive HIF-1α signaling promotes the expansion and effector function of CX3CR1^+^ ‘CTL-like’ exhausted T cells, while promoting the progression of Tpex cells into dysfunctional Tex cells. Of note, in our experiments, we depleted CD4^+^ T cells to ensure persistent infection and T cell exhaustion. Although the depletion of CD4^+^ T cells does not affect Tpex cells directly, the loss of T cell help curtails the population of CX3CR1^+^ exhausted T cells and promotes the conversion of Tpex cells into dysfunctional T cells^55^. Since we depleted CD4^+^ T cells, we may have skewed the differentiation of Tpex into terminally exhausted T cells, which could explain the discrepancy between our study and previous findings^50,52^.

The most prominent HIF-1α gene expression signature across cells and tissues involves glucose metabolism^56–58^. Likewise, single-cell transcriptomics of exhausted T cells also associated the HIF-1α-dependent gene signature with glucose metabolism, including several rate-limiting enzymes of the glycolytic pathway. Thus, it is tempting to speculate that HIF-1α promotes T cell exhaustion via metabolic reprogramming from mitochondrial respiration to aerobic glycolysis. This notion is supported by recent studies demonstrating a decisive role of TGF-β in maintaining Tpex cells^22,59,60^. TGF-β inhibits the transition of Tpex into terminally differentiated Tex cells by suppressing *mechanistic target of rapamycin* (mTOR) and glycolytic reprogramming^22,59,61^. Because mTOR also upregulates HIF-1α expression in activated T cells^48,57,59^, we hypothesize that TGF-β maintains the stemness of Tpex cells by restricting glycolysis via the AKT–mTOR–HIF-1α signaling axis. Additional evidence that limiting glycolytic reprogramming prevents the functional exhaustion of T cells comes from studies using pharmacological strategies^22,62–66^. We here show that short-term treatment with 2-DG reduced the expression of terminal differentiation markers and increased the persistence of T cells in models of chronic viral infection and cancer. These results are consistent with recent reports showing that direct^62–64^ or indirect restriction of glycolysis by inhibiting AKT^65^, mTOR^22^ or calcium signaling^66^ increased T cell longevity and effector function.

Collectively, our findings provide a rationale for manufacturing exhaustion-resistant T cell products for clinical use. Since efficient mitochondrial respiration is crucial for the stemness of exhausted T cells, pharmacological and genetic approaches to enhance their mitochondrial fitness and/or restrict their glycolytic metabolism are promising metabolic intervention strategies to maintain (or reinvigorate) their functionality during cancer immunotherapy.

## Methods

### Mice

All mice were bred and maintained under specific pathogen-free conditions in the Center for Experimental Medicine (ZEMM) or the Institute for Systems Immunology at the Julius-Maximilians University of Würzburg. Mice were housed in individually ventilated cages on a 12/12 h light/dark cycle at 40-60% humidity and 20-24 °C. Mice had access to standard chow (Ssniff; cat# V1534) and autoclaved water *ad libitum* and health status of the animals was inspected daily by the responsible caretakers. The hygiene status of the sentinel mice was monitored quarterly according to the FELASA guidelines. Both male and female mice between 8 and 24 weeks of age at the time of the experiment were used for the in vitro experiments described in this study. For chronic LCMV infection models, male mice between 8 and 12 weeks of age were used. All protocols used for animal experimentation were approved by the animal ethics/welfare committee of the government of Lower Franconia, Germany. C57BL/6 (JAX strain 000664), CD45.1^+^ (strain 002014), P14 (strain 037394), OT-1 (strain 003831), Ubi-GFP (strain 004353), tdTomato^67^, mito-Dendra2 (strain 018397), *Hif1a*^fl/fl^ (strain 007561), *Tfam*^fl/fl^ (strain 026123), *Rag1*^–/–^ (strain 002216) and *Cd4*^Cre^ mice (strain 017336) were purchased from the Jackson Laboratories (JAX) and/or maintained at our institution. *Slc25a3*^fl/fl^ (mPiC) mice^29^ and *Nfatc1*^fl/fl^ mice^68^ were kindly provided by Jeffery D. Molkentin (Cincinnati Children’s Hospital Medical Center, OH, USA) and Anjana Rao (La Jolla Institute for Immunology, CA, USA). All animals used in this study were on a C57BL/6 genetic background.

### In vitro T cell cultures and cell lines

For in vitro cultures, murine CD8 + T cells from male and female mice were isolated from single cell suspension of lymph nodes and spleen by negative selection using the MojoSort Mouse CD8 T cell isolation kit (BioLegend). Alternatively, CD8^+^ T cells were labeled with anti-CD8α-APC (Biolegend, clone 53–6.7) and positively enriched using anti-APC microbeads (Miltenyi Biotech). T cells were cultured in modified RPMI 1640 medium with physiological glucose concentration (100 mg/dL) by diluting standard RPMI 1640 medium (Gibco) with glucose-free RPMI medium (Roth). The medium was supplemented with 10% FBS (Sigma), 50 μM 2-mercaptoethanol (β-ME), 1% penicillin/streptomycin and 1% GlutaMAX-I (all Gibco), unless otherwise stated. Platinum-E retroviral packaging cell line (Cell Biolabs Inc.) was cultured in standard DMEM with 10% FBS (Sigma) and 1% penicillin and streptomycin (Gibco) at 37 °C with 5% CO_2_. MC38^OVA^ and MC38^hCD19^ colon carcinoma cells were grown in DMEM with 10% FBS (Sigma), 2 mM glutamine, 1 mM sodium pyruvate, 1% penicillin/streptomycin, 2 mM Hepes and 0.1 mM non-essential amino acids (all Gibco) at 37 °C with 5% CO_2_.

### T cell activation and differentiation

To activate and differentiate murine CD8^+^ T cells into cytotoxic T cells (CTLs), delta-surface plates (Nunc) were pre-coated with 12 μg/ml polyclonal anti-hamster IgG (MP Biomedicals) for 2 h and washed once with PBS. In 12-well plates, 2 x 10^6^ cells were activated with 0.5 μg/ml of anti-CD3 (clone 145-2C1) together with 1 μg/ml anti-CD28 (clone 37.51, both Bio X Cell). After 2 days of activation, T cells were re-plated with fresh medium containing 10 ng/ml rhIL-2 or a combination of 10 ng/ml IL-7 and IL-15 (all Peprotech) and differentiated at a density of 5 x 10^5^ cells/ml for additional 4 days. For chronic antigenic stimulation in vitro, 2 x 10^6^ cells were activated with 0.5 μg/ml of anti-CD3 and 1 μg/ml anti-CD28 for 2 days followed by anti-CD3 stimulation. A total of 5 x 10^5^ cells/ml were restimulated every other day using 0.5 μg/ml of plate bound anti-CD3 over 6 days. In some experiments, 10 µM N-acetylcysteine (NAC) or 2 mM 2-desoxyglucose (2-DG, both Sigma) were added during cultivation. T cell differentiation was performed in normoxia (21% oxygen) or in hypoxia (2% oxygen) as indicated in the figure legends.

### LCMV production and infection models

LCMV Armstrong (LCMV^ARM^) and clone 13 (LCMV^CL13^) viral stocks were produced with L929/BHK cells. Cells were infected with LCMV in a dose of 0.1 IU/cell for 1 h at RT, before the supernatant was aspirated and replaced. After 48 h and 72 h at 37 °C, the virus-containing supernatant was harvested. For the titration of viral stocks and the measurement of viral loads, a limiting dilution assay was performed. A total of 4 x 10^6^ cells/ml MC57G cells were seeded in 96-well plates and 50 ml of serial virus dilutions from 2 x 10^-2^ to 2 x 10^-6^ were added in eight replicates. Medium was exchanged after 48 h of incubation and after 72 h cells were washed and permeabilized/fixed using Cytofix/Cytoperm kit (BD Biosciences). Cells were stained with VL-4 hybridoma cell line supernatant containing LCMV NP-specific antibodies for 60 min at RT. As a secondary antibody an anti-rat IgG AF488 (Invitrogen) was used. Virus titers were calculated by counting wells with infected cells using Axio Vert.A1 epifluorescence microscope (Zeiss). Prior to LCMV infection (d-1), mice were injected intraperitoneally (i.p.) with 200 µg anti-CD4 monoclonal depletion antibody (clone GK1.5, Bio X Cell). For LCMV^ARM^ infections, mice were infected i.p. with an infectious dose of 2 x 10^5^ IU. LCMV^CL13^ virus stocks were diluted in sterile PBS before intravenous (i.v.) injection of an infectious dose of 4 x 10^6^ IU.

### Adoptive T cell transfers

Purified P14 CD8^+^ T cells were transferred i.v. in sterile PBS into recipient mice. Depending on the experiment, a total of 3–6 x 10^3^ P14 T cells were transferred 1 d before infection. In competitive co-transfer experiments, GFP and tdTomato-expressing WT and transgenic P14 T cells were mixed in a 1:1 ratio before transfer. In some cases, GFP^+^ andTomato^+^ P14 T cells were also labeled with Cell Trace Violet (Thermo Fischer Scientific) before transfer. 2-DG-treated and control P14 T cells were activated with 0.5 μg/ml of anti-CD3 (clone 145-2C1) and 1 μg/ml anti-CD28 (clone 37.51, both Bio X Cell) in presence of 10 ng/ml rhIL-2 (Peprotech) and cultivated for 3–4 days in vitro. A total of 1 × 10^6^ control and 1 × 10^6^ 2-DG-treated P14 T cell were co-transferred i.v. into LCMV^CL13^ infected mice 7 days post-infection (d.p.i.).

### Tumor models

Ovalbumin (OVA) and human CD19-expressing MC38 colon adenocarcinoma (MC38^OVA^ and MC38^hCD19^) cells were grown at 37 °C and 5% CO_2_ in standard DMEM medium supplemented with 10% FBS (Sigma), 2 mM glutamine, 1 mM sodium pyruvate, 1% penicillin/streptomycin, 2 mM Hepes and 0.1 mM non-essential amino acids (NEAA, all Gibco). 5 x 10^5^ MC38^OVA^ cells were subcutaneously (s.c.) injected into the flanks of 8-12 weeks old C57BL/6 mice. Six days after tumor inoculation, mice were irradiated sublethally (6 Gy). The following day, mice were injected intravenously (i.v.) with 2 x 10^6^ OT-I CD8^+^ T cells that were isolated from the spleen and peripheral lymph nodes of OT-1 mice and stimulated with 0.5 μg/ml anti-CD3 (clone 145-2C1), 1 μg/ml anti-CD28 antibodies (clone 37.51, both Bio X Cell) and 10 ng/ml rhIL-2 (Peprotech) for 72 h with or without 1 mM 2-desoxyglucose (2-DG, Sigma). Tumor size was assessed every day using a caliper. For some experiments, tumor tissue was harvested and digested with 1 mg/ml collagenase type I and 100 μg/ml DNase I (both Roche) and T-cell infiltration analyzed by flow cytometry. Alternatively, *Rag1*^–/–^ mice were s.c. injected with 1 × 10^6^ MC38^hCD19^. After 7 days, 2 x 10^6^ anti-hCD19 CAR T cells^33^ treated with and without 2 mM 2-DG for 2 days were adoptively transferred i.v.

### Flow cytometry

Flow cytometric staining was performed as previously described (Vaeth et al., 2019). Briefly, cells were stained with Fixable Viability Dye eFluor 780 (eBioscience) for 10 min in PBS at RT together with an anti-FcgRII/FcgRIII antibody (clone 2.4G2; Bio X Cell) to prevent unspecific binding. After washing, surface antigens were stained with fluorophore-conjugated antibodies in PBS containing 0.5% BSA for 20 min at RT in the dark. For intracellular cytokine staining, cells were stimulated with 1 μM ionomycin (BioMol) and 30 nM phorbol-12- myristat-13-acetate (PMA, Sigma) or with 1 μg/ml of anti-CD3 (clone 145-2C1) together with 1 μg/ml anti-CD28 (clone 37.51, both Bio X Cell) in the presence of 2 μg/ml brefeldin A and 2 μM monensin (both eBioscience) for 4 h at 37 °C. After surface staining, cells were fixed with IC-fixation buffer (eBioscience) and intracellular cytokines were stained using 1x permeabilization buffer (both eBioscience) for 40 min at RT. A complete list of flow cytometry antibodies can be found in Supplementary Table S2. For the quantification of the mitochondrial content and membrane potential, T cells were loaded with 500 nM MitoTracker DeepRed and 2 nM Tetramethylrhodamin-Ethylester (TMRE) (both Invitrogen). As a control for TMRE, cells were pre-treated with 20 µM Trifluoromethoxy carbonylcyanide phenylhydrazone (FCCP, Cayman Chemicals) for 15 min to depolarize the membrane potential. For intracellular ROS determination, MitoSOX and CellROX fluorescent dyes (both ThermoFischer Scientific) were diluted in HBSS (Gibco) buffer and culture medium, respectively. Cells were incubated with the probes for 30 min at 37 °C and washed twice with PBS before analysis. To analyze T cell proliferation, T cells were loaded with 5 μM CellTrace Violet (ThermoFischer Scientific) according to the manufacturer’s instructions. To measure HIF-1α protein expression, stimulated T cells were fixed with the Foxp3/TF staining buffer set (eBioscience) for 30 min at 4 °C and stained intranuclearly with rabbit polyclonal anti-mouse HIF-1α (Cell Signaling) o/n in permeabilization buffer (eBioscience). After washing, the primary antibody was detected using a donkey polyclonal anti-rabbit IgG secondary antibody conjugated to Alexa Fluor 647 (Invitrogen) diluted 1:1000 in permeabilization buffer for 30 min. All sample acquisition was performed with BD Celesta (BD Biosciences) or Attune Nxt (ThermoFischer Scientific) flow cytometers and further analyzed with the FlowJo software (Tree Star). Gating strategies for all flow cytometric analyzes in this study can be found in Supplementary Fig. 10.

### Retroviral transduction

Retroviral infection of primary T cells was performed as described previously (Vaeth et al., 2017a). Briefly, Platinum-E retroviral packaging cells were transfected transiently with modified pMIG retroviral plasmids (Addgene, #9044) or a second-generation anti-hCD19 CAR construct (MSCV-myc-CAR2A-Thy1.1)^33^ using the GeneJet reagent (SignaGene). The transfection medium was replaced 24 h later with standard DMEM medium and the supernatant containing retroviral particles was collected 2 and 3 days after transfection. CD8 + T cells were isolated by negative selection using the MojoSort Mouse CD8^+^ T cell isolation kit (BioLegend) and cultivated as described above. 24 h after activation, the medium of the T cells was replaced by retroviral supernatant, and the cells were transduced by spin-infection (2.500 rpm, 30 °C, 90 min) in the presence of 10 mg/ml polybrene (SantaCruz). After transduction, cells were incubated at 37 °C for 4 h before the viral supernatant was removed and replaced by fresh medium. After 2 days of stimulation, transduced T cells were rested for additional 2 days in fresh medium containing 10 ng/ml rhIL-2 (Peprotech) before 1 x 10^5^ pMIG-empty vector (Ametrine^+^) and 1 x 10^5^ pMIG-SLC25A3 (GFP^+^) expressing P14 T cells were co-transferred into LCMV^CL13^ infected recipient mice or as described above. T cells transduced with the anti-hCD19 CAR construct^33^ were cultivated for an additional 2 days in the presence or without 2 mM 2-DG (Sigma) before adoptive transfer into MC38-hCD19 tumor-bearing mice.

### Mitochondrial turnover measurements

Mito-Dendra2 mice^27^ were infected with LCMV^CL13^ as described above. 14–16 days post infection, splenocytes were harvested and illuminated with LED blue light using a long-pass filter ( ~ 405 nm) for 30 seconds to photoconvert the mitochondrially localized Dendra2 protein. Afterwards, splenocytes were labeled with cell trace violet (CTV) (ThermoFischer Scientific) and cultured for 48 h with 10 ng/ml rhIL-2 (Peprotech) in vitro. Mitochondrial regeneration capacity was calculated based on the reduction of geometric mean fluorescence intensity (gMFI) value of mito-Dendra2-red: (gMFI_0h_-gMFI_48h_)/gMFI_0h_.

### Seahorse extracellular flux analysis

Mitochondrial respiration and lactate secretion of T cells was measured as their oxygen consumption rate (OCR) and glycolytic proton efflux rate (glycoPER), respectively, using an oxygen-controlled XFe96 extracellular flux analyzer (Seahorse Bioscience). XFe96 cell culture microplates (Agilent) were pre-coated with 22 μg/ml Cell-Tak (Corning) and 1-2 x 10^5^ T cells per well were attached in 2–8 replicates in Seahorse XF RPMI medium (Agilent) supplemented with 2 mM L-glutamine (Gibco), 1 mM sodium pyruvate (Sigma) and 10 mM D-glucose (Sigma). After 1 h incubation in a CO_2_-free incubator at 37 °C, glycolytic and mitochondrial stress tests were performed according to the manufacturer’s recommendation. In brief, for assessing glycolysis, basal extracellular acidification rate (ECAR) was measured followed by the addition of 0.5 μM rotenone (AdipoGen) and 0.5 μM antimycin A (Sigma) to inhibit mitochondrial complex 1 and 3, respectively. At the end of the measurement, 50 mM 2-DG (Sigma) was added to completely block glycolysis. To analyze mitochondrial respiration, basal oxygen consumption was measured followed by the addition of 2 μM oligomycin (Cayman Chemicals), an ATP synthase inhibitor, 1 μM of the protonophore carbonyl cyanide-*4*-(trifluoromethoxy)-phenylhydrazone (FCCP, Cayman Chemical) and 0.5 μM rotenone (AdipoGen) together with 0.5 μM antimycin A (Sigma). The basal oxygen consumption was calculated by subtracting the OCR after rotenone and antimycin A treatment from the OCR before oligomycin treatment. The maximal OCR was calculated by subtracting the OCR after rotenone and antimycin A treatment from the OCR measured after the addition of FCCP.

### Metabolomic profiling

To analyze ^12^C and ^13^C polar metabolites, CD8^+^ T cells were activated in RPMI containing 1 g/l ^12^C-glucose as described above. After 2 days of stimulation and resting o/n in fresh medium containing 10 ng/ml rhIL-2 (Peprotech), T cells were labeled in modified RPMI 1640 containing 1 g/l ^13^C_6_-glucose (Sigma). After washing with pre-warmed PBS, metabolite extraction was performed in ice-cold 80% methanol containing 1 μM of ^13^C5-d5-^15^N glutamic acid, 1 μM d7-^15^N4 arginine, 1 μM d27 myristic acid and 1 μM d12 glucose as internal standards. Following centrifugation with 20.000 *g* for 10 min at 4 °C, the supernatant containing polar metabolites was transferred to a new tube and stored at −80 °C. The pellet was used for protein quantification as an internal normalization method. 10 μl sample lysate was loaded into a Dionex UltiMate 3000 LC System (Thermo Scientific) equipped with a C-18 chromatography column (Acquity UPLC -HSS T3 1. 8 μm; 2.1 x 150 mm, Waters) coupled to a Q Exactive Orbitrap mass spectrometer (Thermo Scientific) operating in negative ion mode. A step gradient was carried out using solvent A (10 mM TBA and 15 mM acetic acid) and solvent B (100% methanol). The gradient started with 0% of solvent B and 100% solvent A and remained at 0% B until 2 min post injection. A linear gradient to 37% B was carried out until 7 min and increased to 41% until 14 min. Between 14 and 26 minutes the gradient increased to 100% of B and remained at 100% B for 4 minutes. At 30 min the gradient returned to 0% B. The chromatography stopped at 40 min. The flow was kept constant at 250 μl/min and the column was placed at 25 °C throughout the analysis. The MS operated in full scan mode (m/z range: [70–1050]) using a spray voltage of 3.2 kV, capillary temperature of 320 °C, sheath gas at 40.0, auxiliary gas at 10.0. The AGC target was set at 3e6 using a resolution of 140.000, with a maximum IT fill time of 512 ms. Data collection was performed using the Xcalibur software (Thermo Scientific) and analyzed by integrating the peak areas using the El-Maven to Polly data processing engine (Elucidata). Cellular sphingolipids were analyzed after extraction with methanol:chloroform (2:1) using a 1290 Infinity II HPLC coupled with a 6495 C triple-quadrupole mass spectrometer (Agilent Technologies) as previously described (Naser et al., 2020). Alternatively, water-soluble metabolites were extracted with 500 μL ice-cold MeOH/H_2_O (80/20, v/v) containing 0.01 μM lamivudine and 10 µM each of D4-succinate, D5-glycine, D2-glucose, and 15N-glutamate as standards (Sigma-Aldrich). After centrifugation of the resulting homogenates, supernatants were transferred to an RP18 SPE (50 mg/1 mL tubes, Phenomenex) that had been activated with 0.5 mL CH_3_CN and conditioned with 0.5 mL of MeOH/H2O (80/20, v/v). The eluate of RP18 SPE-column was evaporated in a SpeedVac (Thermo Fisher Scientific). Dry sample extracts were re-dissolved in 100 μL 5 mM NH_4_OAc in CH_3_CN/H_2_O (50/50, v/v). 15 μL supernatant was transferred to LC-vials. For LC/MS analysis 3 μL of each sample was applied to a XBridge Premier BEH Amide (2.5 μm particles, 100 x 2.1 mm) UPLC-column (Waters). Metabolites were separated with Solvent A, consisting of 5 mM NH_4_OAc in CH_3_CN/H_2_O (40/60, v/v) and solvent B consisting of 5 mM NH_4_OAc in CH3CN/H2O (95/5, v/v) at a flow rate of 200 μL/min at 45 °C by LC using a DIONEX Ultimate 3000 UHPLC system (Thermo Fisher Scientific). A linear gradient starting after 2 min with 100% solvent B decreasing to 0% solvent B within 23 min, followed by 17 min 0% solvent B and a linear increase to 100% solvent B in 1 min. Recalibration of the column was achieved by 7 min pre-run with 100% solvent B before each injection. HPLC-MS solvents, NH_4_OAc, standards and reference compounds were purchased from Merck. MS-analyzes were performed on a high-resolution Q Exactive mass spectrometer equipped with a HESI probe (Thermo Fisher Scientific) in alternating positive and negative full MS mode with a scan range of 69.0–1000 m/z at 70 K resolution and the following ESI source parameters: sheath gas: 30, auxiliary gas: 1, sweep gas: 0, aux gas heater temperature: 120 °C, spray voltage: 3 kV, capillary temperature: 320 °C, S-lens RF level: 50. XIC generation and signal quantitation was performed using TraceFinder V 3.3 (Thermo Fisher Scientific) integrating peaks which corresponded to the calculated monoisotopic metabolite masses (MIM + / − H + ± 3 mMU).

### Bulk RNA-sequencing

For bulk RNA-sequencing (RNA-seq), naive T cells of *Slc25a3*^fl/fl^*Cd4*^Cre^ or WT mice were left unstimulated, stimulated with anti-CD3/CD28 for 2 days or differentiated for 6 days into CTLs. For each condition, 1×10^6^ cells were directly harvested in RNAprotect Cell reagent and stored at -80 °C o/n before total RNA was extracted using the RNeasy Plus Micro Kit (both Quiagen). RNA quality and quantity were checked using a 2100 Bioanalyzer with the RNA 6000 Nano kit (Agilent Technologies) and only samples with RIN > 7 were used. cDNA libraries suitable for sequencing were prepared from 500 ng of total RNA with TruSeq mRNA Stranded Library Prep Kit from Illumina according to the manufacturer’s instructions (1/2 volume). Libraries were quantified by QubitTM 3.0 Fluometer (Thermo Scientific) and quality was checked using 2100 Bioanalyzer (Agilent) with High Sensitivity DNA kit (Agilent). 0.5 ng of each library was subjected to a tagmentation-based protocol (Nextera XT, Illumina) using a quarter of the recommended reagent volumes. Libraries were quantified again by QubitTM 3.0 Fluometer (Thermo Scientific) and quality was checked using 2100 Bioanalyzer with High Sensitivity DNA kit (Agilent) before pooling. In both experiments, sequencing of pooled libraries, spiked with 1% PhiX control library, was performed at 19-36 million reads/sample in single-end mode with 75 nt read length on the NextSeq 500 platform (Illumina) with 1 High Output Kit v2.5. Demultiplexed FASTQ files were generated with bcl2fastq2 v2.20.0.422 (Illumina). To assure high sequence quality, Illumina reads were quality and adapter trimmed via Cutadapt [1] version 2.5 using a cutoff Phred score of 20 in NextSeq mode and reads without any remaining bases were discarded. Processed reads were subsequently mapped to the mouse genome (GRCm38.p6 primary assembly and mitochondrion) using STAR v2.7.2b with default parameters based on RefSeq annotation version 108.20200622 for GRCm38.p6 [2]. Read counts on the exon level summarized for each gene were generated using featureCounts v1.6.4 from the Subread package [3]. Multi-mapping and multi-overlapping reads were counted non-strand-specific with a fractional count for each alignment and overlapping feature. The count output was utilized to identify differentially expressed genes using DESeq2 [4] version 1.24.0. Read counts were normalized by DESeq2 and fold-change shrinkage was applied by setting the parameter “betaPrior=TRUE”. Differences in gene expression were considered significant if padj <0.05. The DEseq2 data was further analyzed by gene set enrichment analysis (GSEA) and visualized in Cytoscape using the plugin ErichmentMap in edge cutoff 0.5. Pathways were filtered and displayed when *p* value < 0.005 and Q value < 0.1. A complete list of all gene sets used in this study can be found in Supplementary Table S1.

### Single-cell RNA sequencing

CD8^+^ PD-1^+^ CD44^+^ (Fig. 1) or CD8^+^ tdTomato^+^ (HIF-1α-deficient) and CD8^+^ GFP^+^ tdTomato^+^ (WT) P14 T cells (Fig. 5) were FACS-sorted from the spleen or LNs of chronically infected mice using a FACSAria III cell sorter (BD Biosciences). To multiplex different samples for scRNA sequencing, single-cell suspensions after FACS sorting were labeled with different TotalSeqA antibodies (Biolegend). Each sample was labeled with one specific hashtag (1:400 dilution) and CITE-seq antibodies (1:800 dilution, all Biolegend). After labeling for 30 min at 4 °C, samples were washed three times and multiplexed in equal cell numbers at a density of 10^6^ cells/ml. Singe cells were encapsulated into droplets with the Chromium^TM^ Controller (10x Genomics) and processed following the manufacturer’s specifications. Transcripts captured in all the cells encapsulated with a bead were uniquely barcoded using a combination of a 16 bp 10x Barcode and a 10 bp unique molecular identifier (UMI). cDNA libraries were generated using the Chromium Single Cell 3’ Library & Gel Bead Kit v2 (10x Genomics) following the detailed protocol provided by the manufacturer. Libraries were quantified by QubitTM 3.0 Fluometer (ThermoFisher) and quality was checked using 2100 Bioanalyzer with High Sensitivity DNA kit (Agilent). Libraries were sequenced with the NovaSeq 6000 platform (S1 Cartridge, Illumina) in 50 bp paired-end mode. The sequencing data was demultiplexed using CellRanger software (version 2.0.2). The reads were aligned to mouse mm10 reference genome using STAR aligner. Aligned reads were used to quantify the expression level of mouse genes and the generation of gene-barcode matrix. Subsequent data analysis was performed using Seurat R package (version 3.2) (Stuart et al., 2019).

### scRNA sequencing analysis

Quality control was performed, and viable cells were selected by excluding cells with number of genes lower than 500 and above 4000 and/or having more than 8% of mitochondrial transcripts (Fig. 1). Alternatively, cells having less than 100 transcripts in total and/or more than 8% of mitochondrial transcripts were removed from the analysis (Fig. 5). To demultiplex hashtags HTODemux function in Seurat package was used with standard parameters. Cross-sample doublet cells were detected based on hashtag signal (TotalSeq A0301 and A0302). Cells that were classified as ‘singlet’ and identified by hashtags were retained and used for downstream analysis. Principle component (PC) analysis was used for dimensionality reduction and a uniform manifold approximation and projection (UMAP) was performed on the first 15 PC dimensions. Cells were assigned to clusters with the FindNeighbors function in Seurat on the same PC dimensions and the UMAP clusters were identified using the function FindClusters with a resolution of 0.5 and 0.4 in Figs. 1 and  5, respectively. Contaminating cell types or proliferating cells were removed from the analysis based on marker genes or *Mki67* gene expression, respectively. WT and HIF-1α-deficient samples (Fig. 6) were identified by TotalSeq A0301 and A0302 Hashtag-antibodies, respectively. Enrichment scores for *mitochondrial respiration chain complex assembly* (gene set M11099), *pentose phosphate pathway* (M1386), and HIF-1α target gene signature (combined M17905 and M2513) were calculated using gene lists with AddModuleScore function from Seurat. Differentially expressed genes between WT and HIF-1α-deficient P14 T cells were identified with the FindMarkers function in Seurat (min.pct = 0.25 and logfc.threshold = 0.25) and then subjected to pathway analysis using the EnrichR website (https://maayanlab.cloud/Enrichr/). The KEGG Pathway 2021 Human database was used as a reference to test for pathway enrichment with standard EnrichR settings. Adjusted *p*-values (padj) comparing gene expression of clusters and samples were calculated with the FindMarker function in Seurat; other *p*-values were calculated using Fisher’s exact test.

### Trajectory analysis and gene expression visualization

Trajectories were predicted using Slingshot 1.4.0 package, using function slingshot with a threshold of 0.001, a stretch of 2 and starting with cluster “Tpex1” based on *Sell* (CD62L) expression^9^. Gene expression was visualized as heat maps, volcano and violin plots using the Seurat package and ggplot2.

### Immunoblotting

Cell lysates were prepared in RIPA buffer supplemented with a complete protease-inhibitor cocktail (Roche) and mixed in equal amounts with Laemmli buffer (Roth) before denaturation at 98 °C for 5 min. Protein quantification was performed with the Pierce 660 nm Protein Assay (Pierce). Protein samples were separated via SDS-PAGE, blotted onto nitrocellulose membranes, blocked with 5% BSA, and incubated with monoclonal rabbit anti-HIF-1α (Cell Signaling Technology, clone D1S7W). Detection was carried out with a goat anti-rabbit horse radish peroxidase (HRP) conjugated secondary antibody (BioRad), visualized with the enhanced chemiluminescent SuperSignal reagent (Pierce). For the detection of PGC-1α, TFAM, and elector transport chain (ETC) complex expression, Tpex and Tex cells were FACS sorted from LCMV^CL13^ infected mice 30 d.p.i. and 5x10^5^ T cells were directly lysed in RIPA buffer. Detection of PGC-1α, TFAM, and ETC complexes was carried out using rabbit anti-PGC-1α (Abcam), rabbit-anti-TFAM (Abcam), and an OxPhos rodent antibody cocktail (Thermo) with goat anti-mouse or rabbit HRP-conjugated secondary antibodies (both BioRad).

### Statistical analysis

The results are shown as mean ± standard error of the mean (SEM). To determine the statistical significance of the differences between the experimental groups two-tailed unpaired or paired Student’s *t* tests, 2-way ANOVA and Mantel-Cox tests were performed using the Prism 9 software (GraphPad). Sample sizes were based on experience and experimental complexity, but no methods were used to determine normal distribution of the samples. Differences reached significance with *p* values < 0.05 (noted in figures as *), *p* < 0.01 (**) and *p* < 0.001 (***). The figure legends contain the number of independent experiments or mice per group that were used in the respective experiments.

### Reporting summary

Further information on research design is available in the Nature Portfolio Reporting Summary linked to this article.

### Supplementary information

Supplementary Information

Peer Review File

Reporting Summary

### Source data

Source Data

## Data Availability

The bulk and single-cell RNA sequencing data generated in this study have been deposited in the Gene Expression Omnibus (GEO) database under accession codes GSE212298, GSE214003, and GSE213847. The remaining data are available within the Article, Supplementary Information, or Source Data file. Source data are provided with this paper.
