# Supplementary material for: Mitochondrial dysfunction promotes the transition of precursor to terminally exhausted T cells through HIF-1α-mediated glycolytic reprogramming
Source: Nat Commun. 2023 Oct 27;14:6858. doi: 10.1038/s41467-023-42634-3 (PMC10611730; doi:10.1038/s41467-023-42634-3)
Supplement: Supplementary file 1 — Supplementary Information [file 41467_2023_42634_MOESM1_ESM.pdf]

Supplementary Information

Mitochondrial dysfunction promotes the transition of precursor to terminally exhausted T cells through HIF-1α-mediated glycolytic reprogramming

Hao Wu[1], Xiufeng Zhao[1], Sophia M. Hochrein[1], Miriam Eckstein[1], Gabriela F. Gubert[1], Konrad Knöpper[1], Ana Maria Mansilla[1], Arman Öner[2], Remi Doucet-Ladevèze[1], Werner Schmitz[3], Bart Ghesquière[4], Sebastian Theurich[5,8], Jan Dudek[6], Georg Gasteiger[1], Alma Zernecke[7], Sebastian Kobold[2,8], Wolfgang Kastenmüller[1] and Martin Vaeth[1*]

[1]Würzburg Institute of Systems Immunology, Max Planck Research Group, Julius-Maximilians University of Würzburg, Würzburg, Germany

[2]Division of Clinical Pharmacology, Department of Medicine IV, Ludwig Maximilians University (LMU) Munich, University Hospital, Munich, Germany.

[3]Department of Biochemistry and Molecular Biology, Theodor Boveri Institute, Biocenter, Julius-Maximilians University of Würzburg, Germany

[4]Laboratory of Applied Mass Spectrometry, Department of Cellular and Molecular Medicine, KU Leuven, Leuven, Belgium and Metabolomics Core Facility Leuven, Center for Cancer Biology, VIB, Leuven, Belgium

[5]Ludwig Maximilians University (LMU) Munich, University Hospital, Department of Medicine III, Munich, Germany and LMU Gene Center, Cancer and Immunometabolism Research Group, Munich, Germany

[6]Comprehensive Heart Failure Center (CHFC), University Hospital, Julius-Maximilians University of Würzburg, Germany

[7]Institute of Experimental Biomedicine, University Hospital Würzburg, Würzburg, Germany

[8]German Cancer Consortium (DKTK), partner site Munich, a partnership between the DKFZ Heidelberg and the University Hospital of the LMU

*Correspondence to Martin Vaeth (martin.vaeth1@uni-wuerzburg.de)

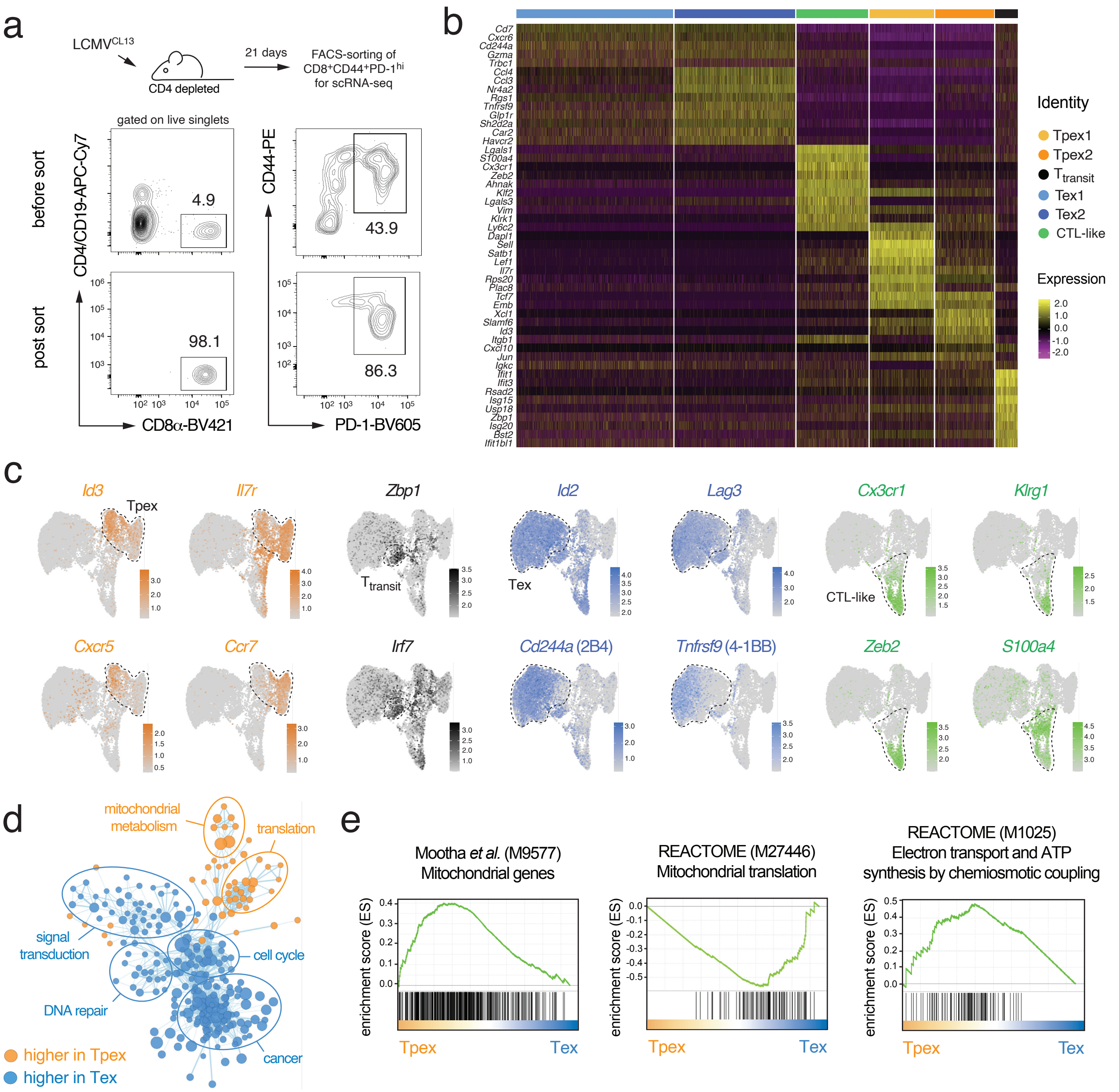

Supplementary Figure 1. Transcriptional and metabolic features of T cell exhaustion. (a) Naïve C57BL/6 mice were chronically infected with the LCMV strain clone 13 (LCMV^{CL13}) and CD8^{+}CD44^{+}PD-1^{hi} T cells were FACS sorted and subjected to single cell (sc) RNA sequencing at day 21 post infection. **(b)** Heat map of marker gene expression associated with the uniform manifold approximation and projection (UMAP) clusters depicted in Fig. 1a. **(c)** Normalised gene expression of *Id3*, *Il7r, Cxcr5, Ccr7, Zbp1, Irf7, Id2, Lag3, Cd244a* (2B4) and *Tnfrsf9* (41-BB) projected onto UMAP clusters. **(d)** Network clustering of publicly available bulk RNA sequencing data of Tpex and Tex cells (Utzschneider *et al*. 2020; GSE142686) using significantly ($p < 0.05$) enriched gene expression signatures. Upregulated gene sets in Tpex and Tex are shown in orange and blue, respectively. **(e)** Gene set enrichment analysis (GSEA) of *Mootha mitochondria* (gene set M9577), *mitochondrial translation* (M27446) and *respiratory electron transport ATP synthesis by chemiosmotic coupling by uncoupling proteins* gene signatures (M1025) in Tpex and Tex cells.

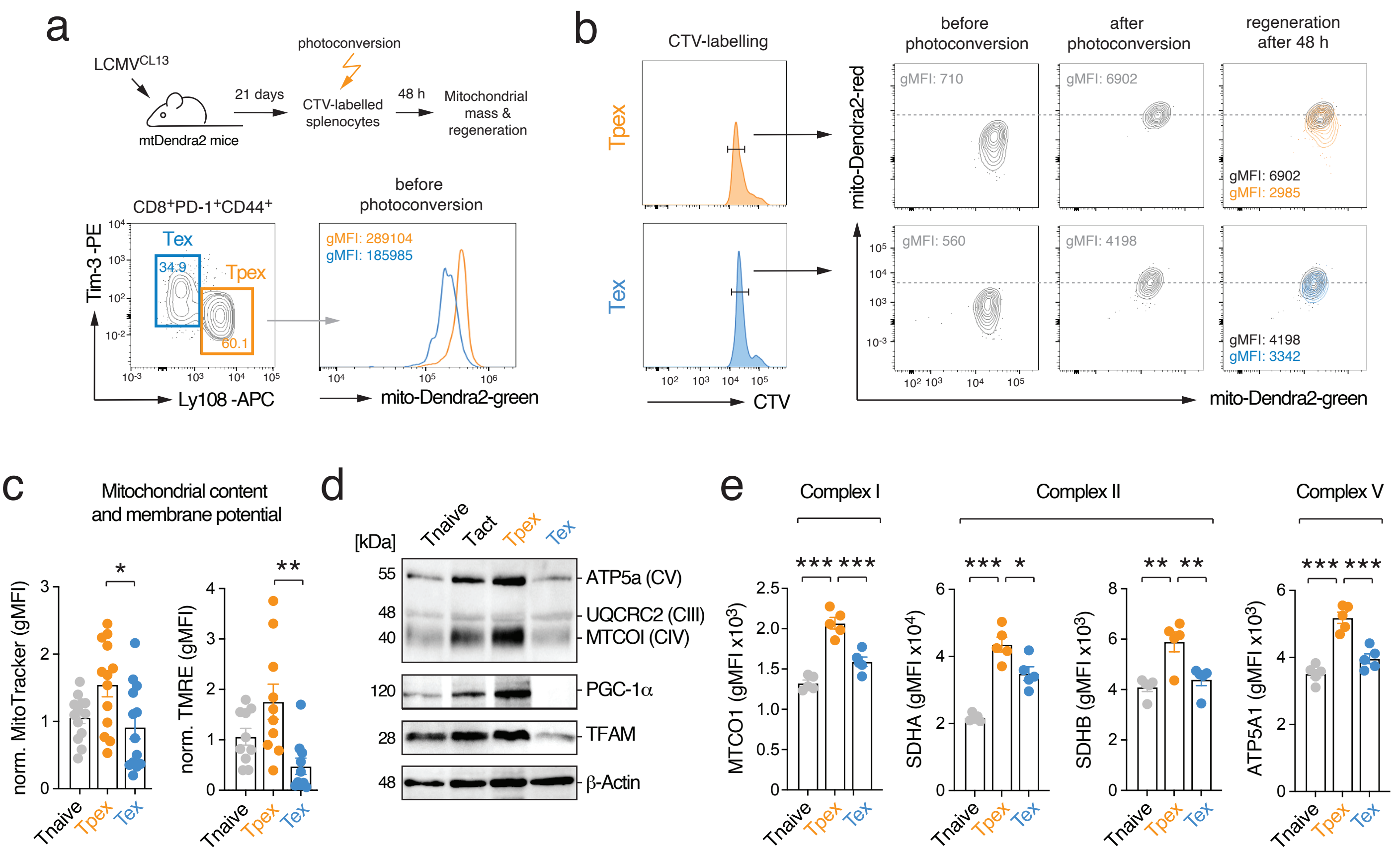

Supplementary Figure 2. Characterization of mitochondria in Tpex and Tex cells. (a) Analysis of mitochondrial mass/volume in Tpex and Tex cells using LCMV^{CL13} infected mito-Dendra2 mice (before photo-conversion). **(b)** Mitochondrial regeneration capacity of non-divided Tpex and Tex cells *ex vivo*. T cells were isolated from LCMV^{CL13} infected mito-Dendra2 mice and photo-converted with 405 nm laser light. Mito-Dendra2-red and mito-Dendra2-green was measured by flow cytometry 48 h after photoconversion. **(c)** Flow cytometric analysis of mitochondrial volume/mass and membrane potential in Tnaive, Tpex and Tex cells using MitoTracker and TMRE probes, respectively; means ± SEM of 9-11 mice. **(d)** Western blot analyses of mitochondrial electron transport chain (ETC) complexes, PGC-1α and TFAM expression in Tnaive, Tact, Tpex and Tex cells isolated from WT mice by FACS sorting 21 days after chronic LCMV infection. **(e)** Analysis of mitochondrial (ETC) complexes by flow cytometry 21 days after LCMV^{CL13} infection; means ± SEM of 4-5 mice. *, p<0.05; **, p<0.01; ***, p<0.001 by unpaired Student's t-test in (c,e).

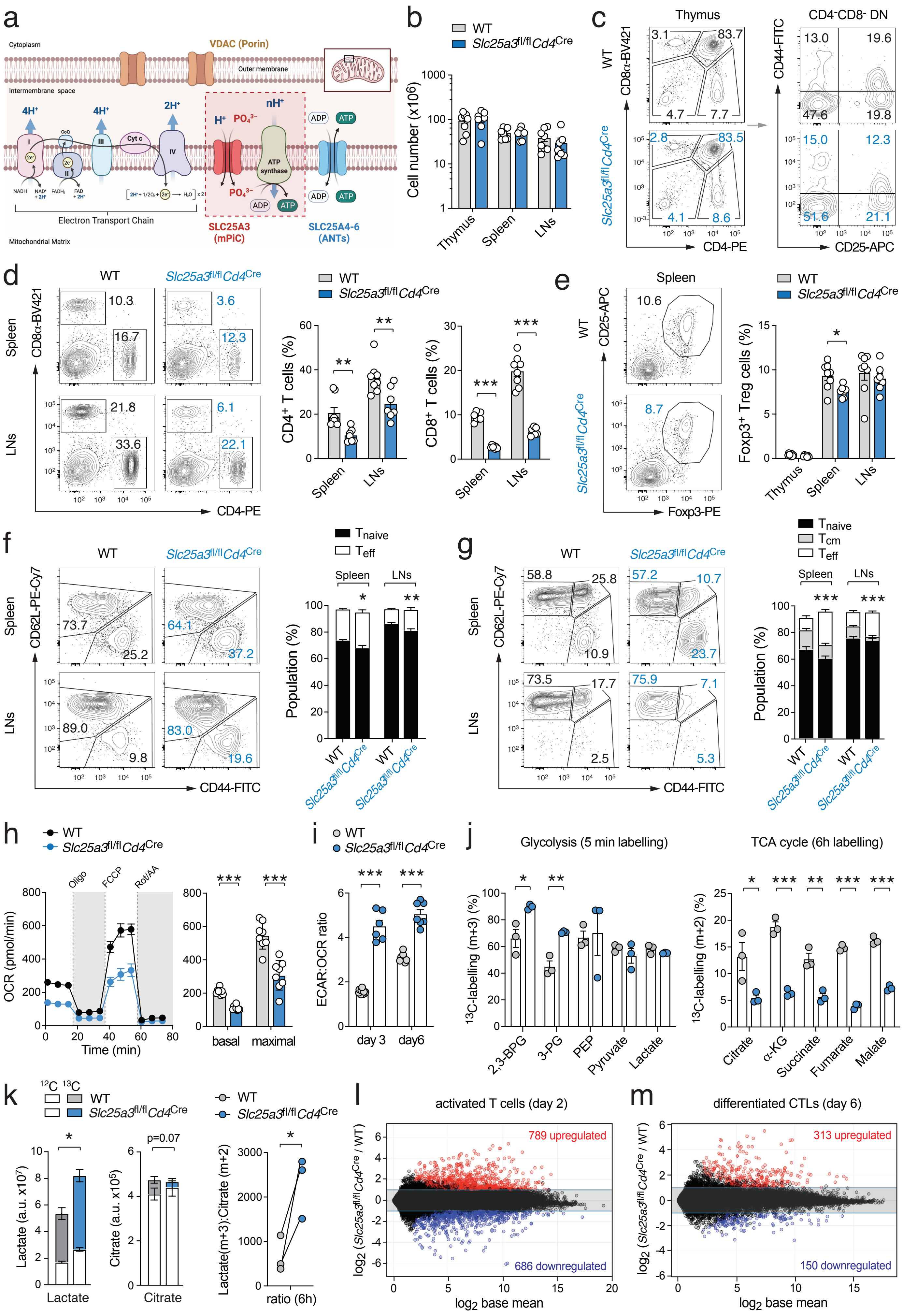

a
Cytoplasm
VDAC (Porin)
Outer membrane
Intermembrane space
Electron Transport Chain
Mitochondrial Matrix
SLC25A3 (mPiC)
SLC25A4-6 (ANTs)
ATP synthase
b
WT
Slc25a3fl/fl Cd4Cre
Cell number (x10⁶)
Thymus
Spleen
LNs
c
Thymus
CD4-CD8- DN
CD8α-BV421
CD4-PE
CD44-FITC
CD25-APC
d
CD4+ T cells (%)
CD8+ T cells (%)
e
Foxp3+ Treg cells (%)
Foxp3-PE
f
CD62L-PE-Cy7
CD44-FITC
Tnaive
Teff
Population (%)
g
Tcm
h
OCR (pmol/min)
Time (min)
Oligo
FCCP
Rot/AA
basal
maximal
i
ECAR:OCR ratio
day 3
day6
j
Glycolysis (5 min labelling)
TCA cycle (6h labelling)
13C-labelling (m+3) (%)
13C-labelling (m+2) (%)
2,3-BPG
3-PG
PEP
Pyruvate
Lactate
Citrate
α-KG
Succinate
Fumarate
Malate
k
Lactate (a.u. x10⁷)
Citrate (a.u. x10⁵)
p=0.07
Lactate(m+3):Citrate (m+2)
ratio (6h)
l
activated T cells (day 2)
789 upregulated
686 downregulated
log2 base mean
m
differentiated CTLs (day 6)
313 upregulated
150 downregulated

Supplementary Figure 3. Immunological characterization of *Slc25a3*$^{fl/fl}$*Cd4*^{Cre} mice and mPiC-deficient CD8$^+$ T cells *in vitro*. (a) Transport of inorganic phosphate (Pi) into the mitochondrial matrix is mediated by the mitochondrial phosphate carrier (mPiC), encoded by the *Slc25a3* gene. **(b-g)** Flow cytometric characterization of mPiC-deficient (*Slc25a3*$^{fl/fl}$*Cd4*^{Cre}) mice. **(b)** Total cell numbers of thymus, spleen and lymph nodes (LNs) of 8-14 weeks old WT and *Slc25a3*$^{fl/fl}$*Cd4*^{Cre} mice; means ± SEM of 7-8 mice. **(c)** Representative flow cytometric analyses of thymic T cell subsets in WT and *Slc25a3*$^{fl/fl}$*Cd4*^{Cre} mice. **(d, e)** Analysis of peripheral CD4$^+$ and CD8$^+$ T cell subsets (d) and Foxp3$^+$ Treg cells (e) in spleens and LNs of WT and *Slc25a3*$^{fl/fl}$*Cd4*^{Cre} mice; means ± SEM of 7-8 mice. **(f, g)** Flow cytometric analysis of CD44$^-$CD62L$^+$ (naïve), CD44$^+$CD62L$^+$ (central memory) and CD44$^+$CD62L$^-$ (effector) T cells of WT and *Slc2a3*$^{fl/fl}$*Cd4*^{Cre} mice; means ± SEM of 7-8 mice. **(h)** Analyses of oxygen consumption rate (OCR) of WT and mPiC-deficient (*Slc25a3*$^{fl/fl}$*Cd4*^{Cre}) cytotoxic lymphocytes (CTLs) at day 6 of culture using a Seahorse extracellular flux analyzer; means ± SEM of 3 mice. **(i)** Ratio of extracellular acidification rate (ECAR) to OCR in WT and mPiC-deficient T cells after anti-CD3/CD28 stimulation *in vitro*; means ± SEM of 3 mice. **(j)** Isotope tracing of glucose-derived metabolites in WT and GLUT3-deficient T cells by liquid chromatography and mass spectrometry (LC/MS) after incubation with ^{13}C-glucose. Analysis of (m+3) glycolytic and (m+2) TCA cycle metabolites in WT and mPiC-deficient T cells after 5 min and 6 h labeling with ^{13}C-glucose. Fractional enrichments of ^{13}C-metabolites are shown as means ± SEM of 3 biological replicates. **(k)** Analysis of ^{12}C and ^{13}C-labelled lactate (m+3) and citrate (m+2) by LC/MS after incubation with ^{13}C-glucose for 6 h. Ratio of ^{13}C-lactate to ^{13}C-citrate in WT and mPiC-deficient T cells; means ± SEM of 3 biological replicates. **(l, m)** MA plots of differentially expressed genes (DEGs) in WT *versus* mPiC-deficient T cells. Naïve T cells were activated with anti-CD3/CD28 for two days (day 2) or differentiated into CTLs for six days (day 6). *, p<0.05; **, p<0.01; ***, p<0.001 by unpaired Student's t-test in (d-k).

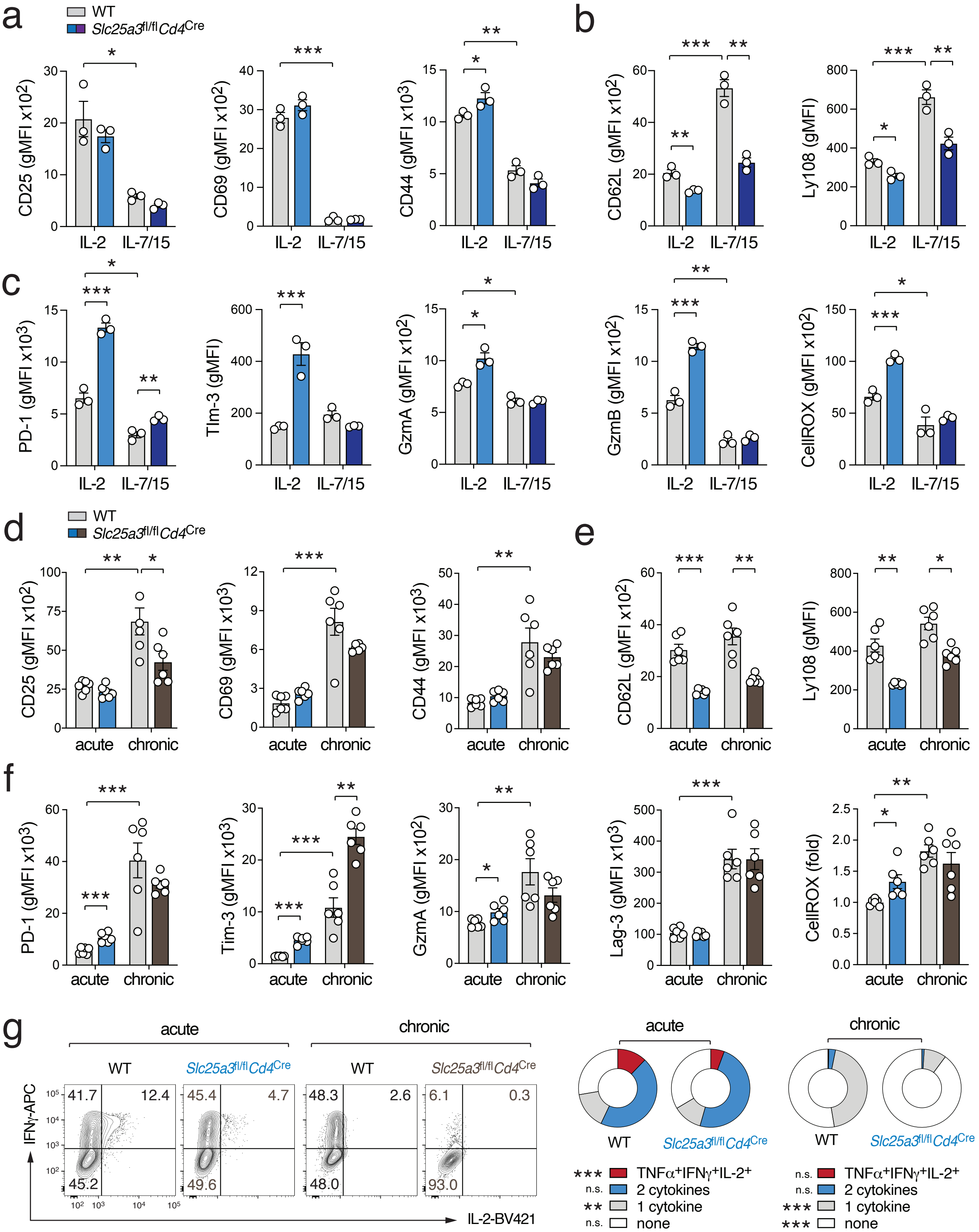

a
WT
Slc25a3fl/flCd4Cre
CD25 (gMFI x10²)
CD69 (gMFI x10²)
CD44 (gMFI x10³)
IL-2
IL-7/15
b
CD62L (gMFI x10²)
Ly108 (gMFI)
c
PD-1 (gMFI x10³)
Tim-3 (gMFI)
GzmA (gMFI x10²)
GzmB (gMFI x10²)
CellROX (gMFI x10²)
d
WT
Slc25a3fl/flCd4Cre
CD25 (gMFI x10²)
CD69 (gMFI x10³)
CD44 (gMFI x10³)
acute
chronic
e
CD62L (gMFI x10²)
Ly108 (gMFI)
f
PD-1 (gMFI x10³)
Tim-3 (gMFI x10³)
GzmA (gMFI x10²)
Lag-3 (gMFI x10³)
CellROX (fold)
g
acute
chronic
WT
Slc25a3fl/flCd4Cre
41.7
12.4
45.2
45.4
4.7
49.6
48.3
2.6
48.0
6.1
0.3
93.0
IFNγ-APC
IL-2-BV421
TNFα+IFNγ+IL-2+
2 cytokines
1 cytokine
none
n.s.

Supplementary Figure 4. Differentiation of WT and mPiC-deficient CD8$^+$ T cells with IL-7 plus IL-15 or chronic antigenic stimulation. (a-c) Expression of activation (a), memory (b) and exhaustion markers (c) on cytotoxic lymphocytes (CTLs) and memory-like T cells differentiated from naïve WT and mPiC-deficient (*Slc25a3*$^{fl/fl}$*Cd4*^{Cre}) T cells. CD8$^+$ T cells were activated with anti-CD3/CD28 for 2 days followed by 4 days resting in IL-2 or a combination of IL-7 and IL-15 to generate CTL and memory-like T cells, respectively; means ± SEM of 3 mice. **(d-f)** Expression of activation (d), memory (e) and exhaustion markers (f) on WT and mPiC-deficient T cells differentiated under acute and chronic antigenic stimulation. CD8$^+$ T cells were activated with anti-CD3/CD28 for 2 days followed by 4 days of resting in IL-2 (acute) or continuous antigenic stimulation with anti-CD3/CD28 over 6 days (chronic); means ± SEM of 6 mice. **(g)** Analysis of polyfunctional TNFα, IFNγ and IL-2 expression by WT and mPiC-deficient T cells differentiated under acute and chronic antigenic stimulation and restimulation with anti-CD3/CD28 for 6 h; means ± SEM of 3 mice. *, $p<0.05$; **, $p<0.01$; ***, $p<0.001$ by unpaired Student's t-test in (a-g).

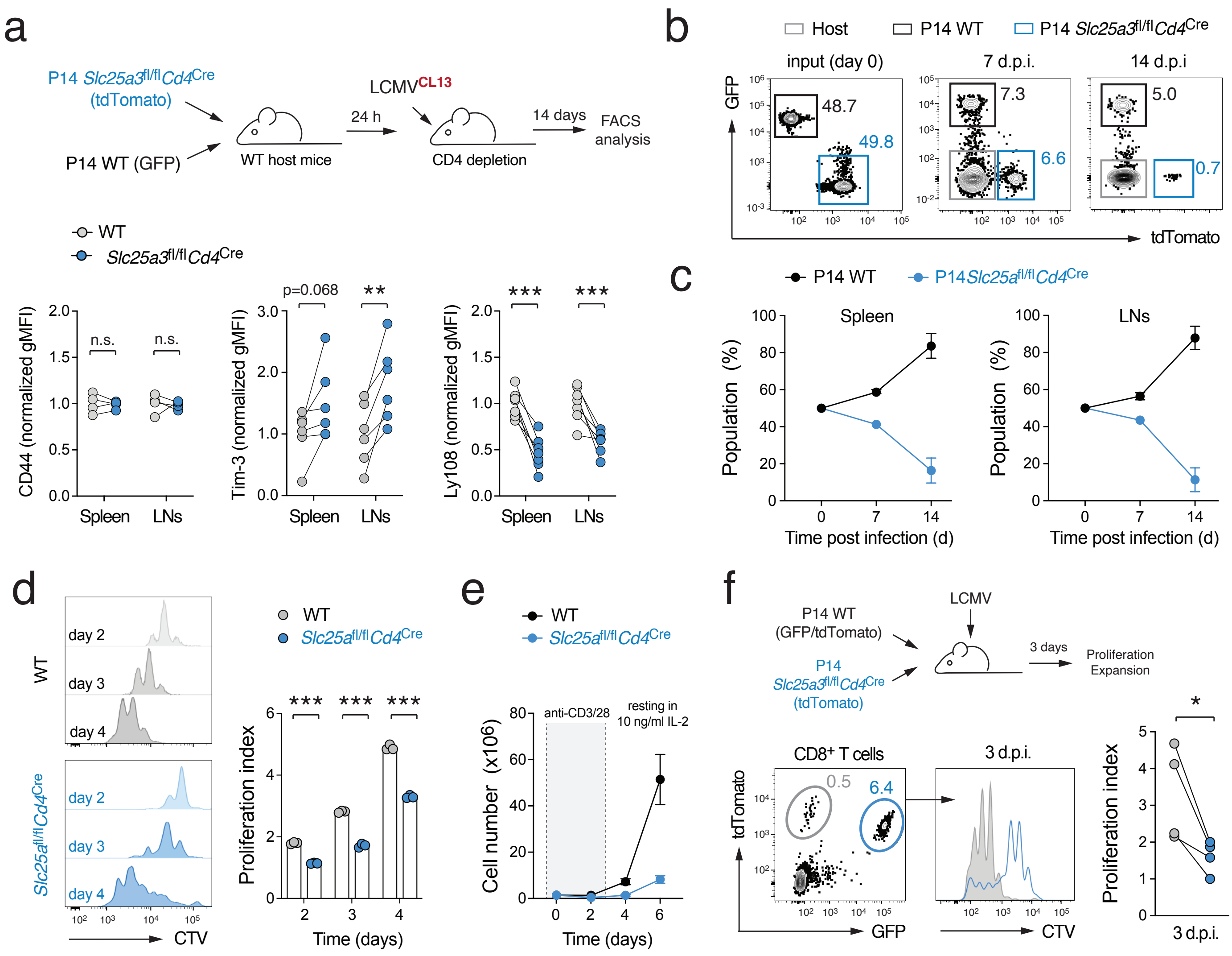

Supplementary Figure 5. Mitochondrial respiration controls the proliferation and clonal expansion of virus-specific T cells *in vivo*. (a) Adoptive co-transfer of naïve GFP$^+$ WT and tdTomato$^+$ mPiC-deficient (*Slc25a3*$^{fl/fl}$*Cd4*^{Cre}) P14 T cells into C57BL/6 mice before infection with LCMV clone 13 (LCMV^{CL13}). Flow cytometric analysis of CD44, Tim-3 and Ly108 expression on donor P14 T cells in the spleen and LNs 14 days post infection (d.p.i.). **(b)** Representative flow cytometric analyses of splenic GFP$^+$ (WT) and tdTomato$^+$ (mPiC-deficient) donor P14 T cells at different timepoints after transfer into LCMV^{CL13} infected mice. **(c)** Relative proportions of WT and mPiC-deficient donor P14 T cells in the spleens and LNs of chronically infected recipient mice; means ± SEM of 4-8 mice per timepoint. **(d)** Proliferation of WT and mPiC-deficient CD8$^+$ T cells by CellTrace Violet (CTV) dilution over a course of 4 days *in vitro*. **(e)** Cellular expansion of WT and mPiC-deficient T cells in vitro; means ± SEM of 3 mice per timepoint. **(f)** Proliferation analysis of WT and mPiC-deficient P14 T cells *in vivo*. Adoptive co-transfer of CTV-labelled GFP$^+$ tdTomato$^+$ WT and tdTomato$^+$ mPiC-deficient P14 T cells into chronically infected C57BL/6 WT host mice. Flow cytometric analysis of proliferation and cellular expansion 3 days after adoptive transfer. *, p<0.05; **, p<0.01; ***, p<0.001 by paired (a and f) and unpaired (d) Student's t-tests.

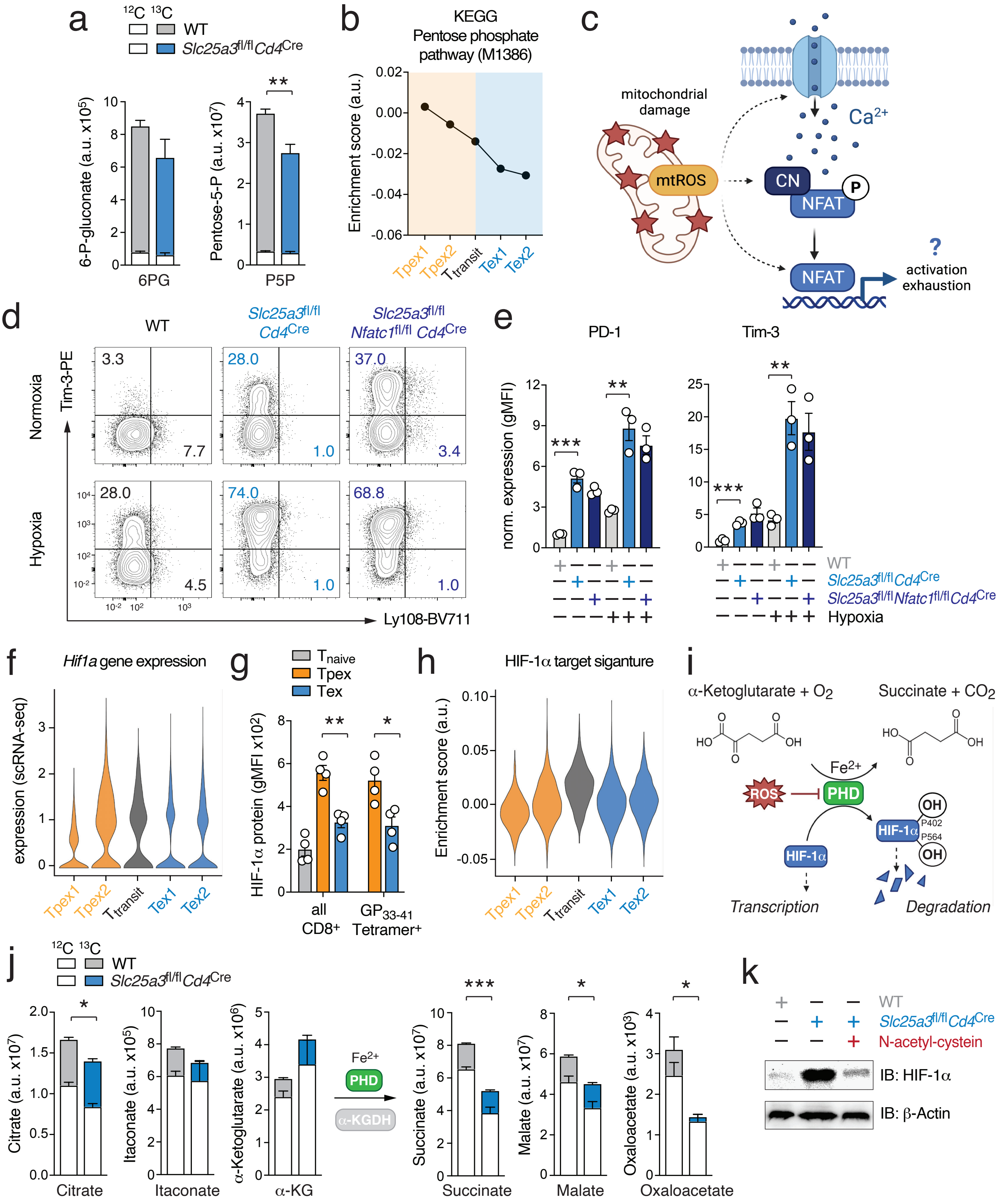

a
12C 13C
WT
Slc25a3fl/fl Cd4Cre
6-P-gluconate (a.u. x10^5)
6PG
Pentose-5-P (a.u. x10^7)
**
P5P
b
KEGG
Pentose phosphate pathway (M1386)
Enrichment score (a.u.)
Tpex1
Tpex2
Ttransit
Tex1
Tex2
c
mitochondrial damage
mtROS
CN
P
NFAT
Ca2+
NFAT
?
activation
exhaustion
d
WT
Slc25a3fl/fl Cd4Cre
Slc25a3fl/fl Nfatc1fl/fl Cd4Cre
Normoxia
Hypoxia
Tim-3-PE
Ly108-BV711
3.3
7.7
28.0
1.0
37.0
3.4
28.0
4.5
74.0
1.0
68.8
1.0
e
PD-1
Tim-3
norm. expression (gMFI)
***
**
***
**
WT
Slc25a3fl/fl Cd4Cre
Slc25a3fl/fl Nfatc1fl/fl Cd4Cre
Hypoxia
f
Hif1a gene expression
expression (scRNA-seq)
Tpex1
Tpex2
Ttransit
Tex1
Tex2
g
Tnaive
Tpex
Tex
HIF-1α protein (gMFI x10^2)
**
*
all CD8+
GP33-41 Tetramer+
h
HIF-1α target siganture
Enrichment score (a.u.)
Tpex1
Tpex2
Ttransit
Tex1
Tex2
i
α-Ketoglutarate + O2
Succinate + CO2
Fe2+
ROS
PHD
HIF-1α
OH
P402
P564
OH
HIF-1α
Transcription
Degradation
j
12C 13C
WT
Slc25a3fl/fl Cd4Cre
Citrate (a.u. x10^7)
*
Citrate
Itaconate (a.u. x10^5)
Itaconate
α-Ketoglutarate (a.u. x10^6)
α-KG
Fe2+
PHD
α-KGDH
Succinate (a.u. x10^7)
***
Succinate
Malate (a.u. x10^7)
*
Malate
Oxaloacetate (a.u. x10^3)
*
Oxaloacetate
k
WT
Slc25a3fl/fl Cd4Cre
N-acetyl-cystein
IB: HIF-1α
IB: β-Actin

Supplementary Figure 6. Terminal differentiation of mPiC-deficient T cells is mediated by ROS and HIF-1α. (a) Isotope tracing of glucose-derived 6-phosphogluconate (6PG) and pentose-5-phosphate (P5P) in WT and mPiC-deficient (*Slc25a3*$^{fl/fl}$*Cd4*^{Cre}) T cells by liquid chromatography and mass spectrometry (LC/MS) after incubation with ^{13}C-glucose for 24 h. Fractional enrichment of ^{13}C-glucose derived metabolites in WT and mPiC-deficient T cells is depicted in grey and blue, respectively. Statistical analysis on total metabolite levels; 5 biological replicates per group. **(b)** Enrichment of pentose phosphate pathway gene expression signatures (gene set ID M1386) in UMAP clusters representing Tpex and Tex cell subsets as shown in Fig. 1a-d. **(c)** Mitochondrial ROS may enhance the Ca^{2+} / calcineurin / NFAT signaling pathway in activated T cells. **(d, e)** Differentiation of WT, mPiC-deficient (*Slc25a3*$^{fl/fl}$*Cd4*^{Cre}) and mPiC/NFATc1 double-deficient (*Slc25a3*$^{fl/fl}$*Nfatc1*$^{fl/fl}$*Cd4*^{Cre}) T cells *in vitro*. Representative flow cytometric analysis (d) and quantification of PD-1 and Tim-3 expression on WT, mPiC-deficient and mPiC/NFATc1 double-deficient T cells (e); means ± SEM of 3 mice. **(f)** Analysis of *Hif1a* gene expression in different Tpex and Tex UMAP clusters analyzed by scRNA sequencing as shown in Fig. 1a-d. **(g)** Quantification of HIF-1α protein expression in Tpex and Tex cells in all $CD8^+$ T cells and LCMV-specific (GP_{33-41} teramer$^+$) T cells in the spleen of WT mice 21 days after LCMV^{CL13} infection; means ± SEM of 4 mice. **(h)** Enrichment analysis of the HIF-1α target gene expression (combined gene sets M17905 and M2513) in Tpex and Tex cell clusters analyzed by scRNA sequencing as shown in Fig. 1a-d. **(i)** ROS prevents HIF-1α protein degradation through oxidative inhibition of prolyl hydoxylases (PHDs). **(j)** Isotope tracing of glucose-derived TCA cycle metabolites in WT and mPiC-deficient T cells after incubation with ^{13}C-glucose for 24 h. Fractional enrichment of ^{13}C-glucose derived metabolites in WT and mPiC-deficient T cells is depicted in grey and blue, respectively. Statistical analysis based on total metabolite levels; 5 biological replicates per group. **(k)** Western blot analysis of HIF-1α protein expression in WT and mPiC-deficient T cells with and without ROS scavenging using N-acetyl cysteine (NAC). *, p<0.05; **, p<0.01; ***, p<0.001 by unpaired Student's t-test in (a), (e), (g) and (j).

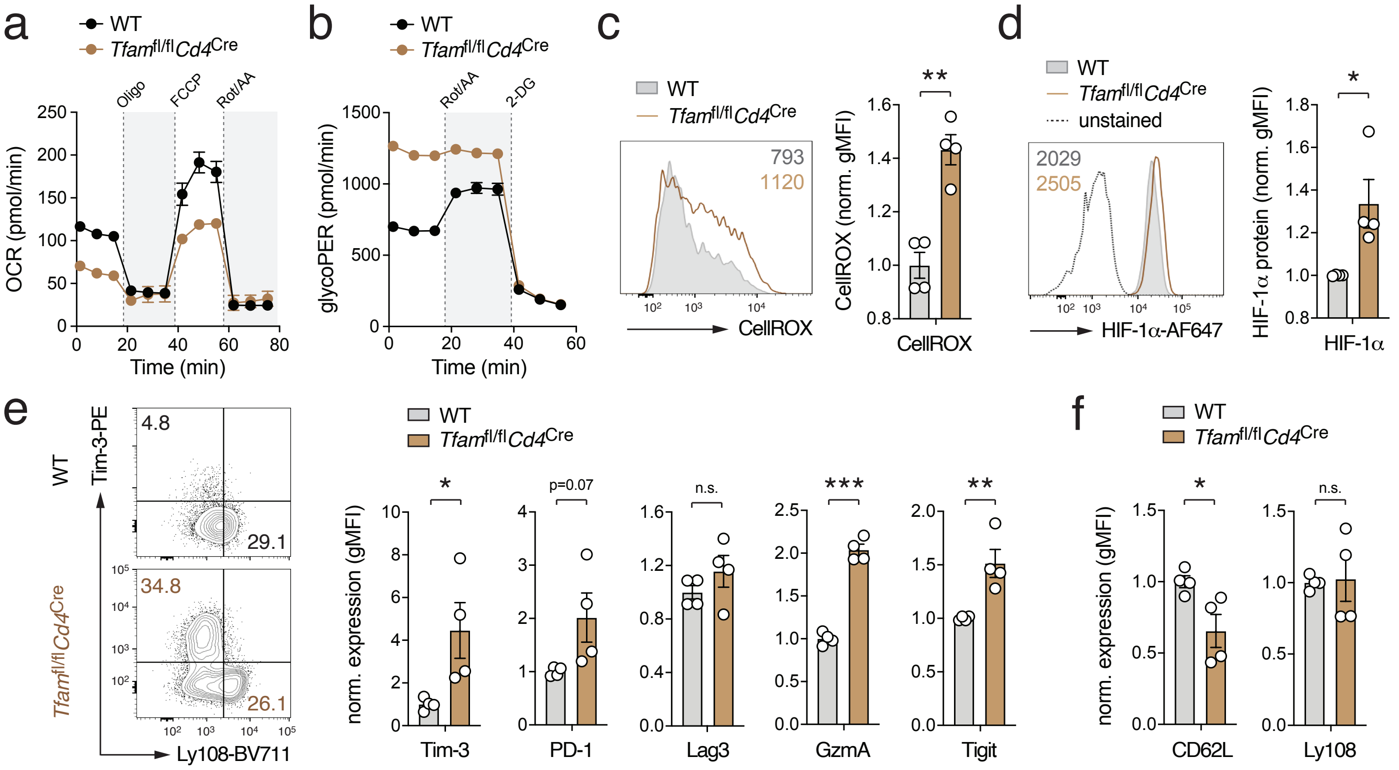

Supplementary Figure 7. Ablation of TFAM in T cells causes metabolic reprogramming, ROS production, HIF-1α stabilization and terminal exhaustion. (a, b) Analyses of oxygen consumption rate (OCR) and glycolytic proton efflux rate (glycoPER) in anti-CD3/CD28 activated WT and TFAM-deficient (*Tfam*$^{fl/fl}$*Cd4*^{Cre}) T cells using a Seahorse extracellular flux analyzer; means ± SEM of 2-3 mice. **(c)** Flow cytometric analysis of cellular ROS production (CellROX) in anti-CD3/CD28 stimulated WT and TFAM-deficient CD8$^+$ T cells; means ± SEM of 4 mice. **(d)** Analysis of HIF-1α protein expression in activated WT and TFAM-deficient CD8$^+$ T cells by flow cytometry; means ± SEM of 4 mice. **(e, f)** Flow cytometric detection of exhaustion (e) and stemness marker (f) expression in WT and TFAM-deficient T cells, means ± SEM of 4 mice. *, p<0.05; **, p<0.01; ***, p<0.001 by unpaired Student's t-test in (c-f).

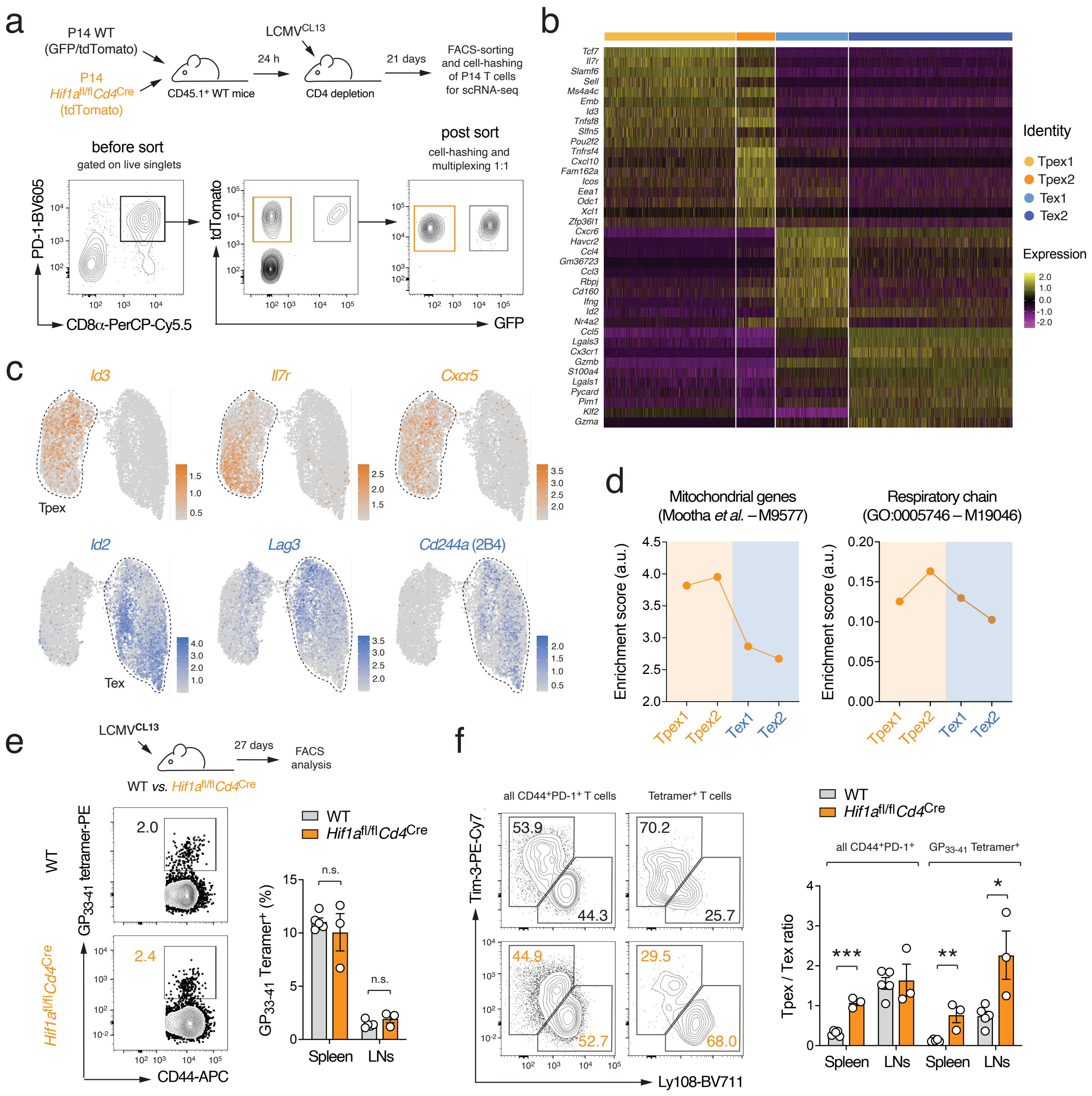

Supplementary Figure 8. HIF-1α controls the exhaustion of virus-specific T cells. (a) Adoptive co-transfer of tdTomato⁺GFP⁺ WT and tdTomato⁺ HIF-1α-deficient (*Hif1a*$^{fl/fl}$*Cd4*^{Cre}) P14 T cells into C57BL/6 mice before LCMV clone 13 (LCMV^{CL13}) infection. 21 days post infection (d.p.i.), donor WT and HIF-1α-deficient P14 T cells were FACS sorted, barcoded and multiplexed in a 1:1 ratio before subjected to scRNA sequencing. **(b)** Heat map of marker gene expression associated with the uniform manifold approximation and projection (UMAP) clusters depicted in Fig. 5a. **(c)** Normalized gene expression of *Id3*, *Il7r, Cxcr5, Id2, Lag3* and *Cd244a* (2B4) projected onto UMAP clusters. **(d)** Enrichment of mitochondrial (gene set ID M9577) and respiratory chain gene expression signatures (M19046) in UMAP clusters representing Tpex and Tex cell subsets as shown in Fig. 5a. **(e, f)** Chronic infection of WT and *Hif1a*$^{fl/fl}$*Cd4*^{Cre} mice with LCMV^{CL13}. **(e)** Analysis of LCMV-specific ($GP_{33\text{-}41}$ tetramer$^+$) T cells in the spleen and LNs of WT and *Hif1a*$^{fl/fl}$*Cd4*^{Cre} mice 27 d.p.i.; means ± SEM of 3-5 mice. **(f)** Flow cytometric analysis of Tpex and Tex cells among CD44$^+$PD-1$^+$ and LCMV-specific ($GP_{33\text{-}41}$ tetramer$^+$) CD8$^+$ T cells in the spleen and LNs of WT and *Hif1a*$^{fl/fl}$*Cd4*^{Cre} mice 14 d.p.i.; means ± SEM of 3-5 mice. *, $p<0.05$; **, $p<0.01$; ***, $p<0.001$ by unpaired Student's t-test in (f).

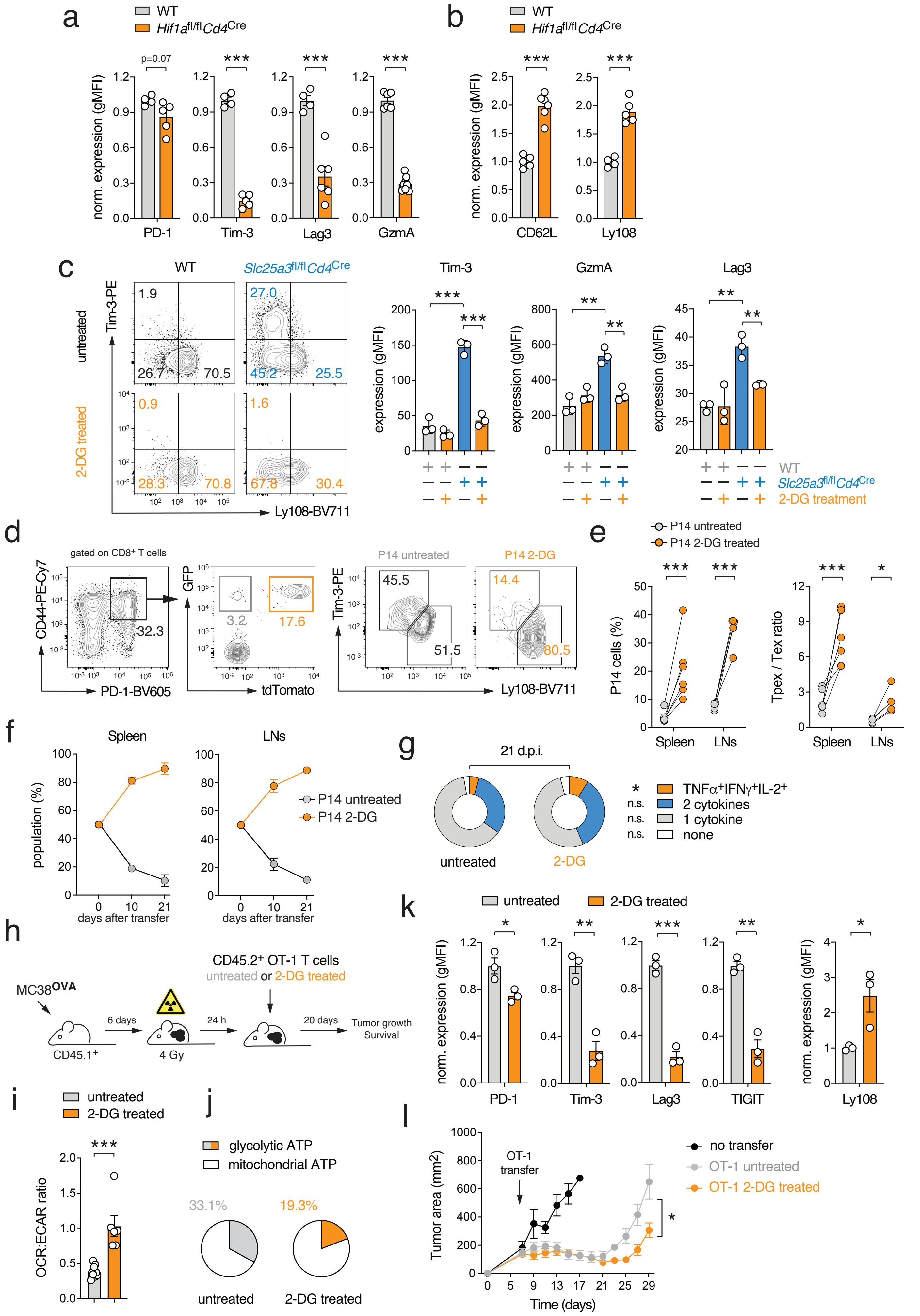
a
WT
Hif1a fl/fl Cd4 Cre
p=0.07
***
norm. expression (gMFI)
PD-1
Tim-3
Lag3
GzmA
b
CD62L
Ly108
c
WT
Slc25a3 fl/fl Cd4 Cre
untreated
2-DG treated
Tim-3-PE
Ly108-BV711
1.9
26.7
70.5
27.0
45.2
25.5
0.9
28.3
70.8
1.6
67.8
30.4
Tim-3
GzmA
Lag3
expression (gMFI)
2-DG treatment
d
gated on CD8+ T cells
CD44-PE-Cy7
PD-1-BV605
32.3
GFP
tdTomato
3.2
17.6
P14 untreated
P14 2-DG
45.5
51.5
14.4
80.5
e
P14 untreated
P14 2-DG treated
P14 cells (%)
Tpex / Tex ratio
Spleen
LNs
f
Spleen
LNs
population (%)
days after transfer
P14 untreated
P14 2-DG
g
21 d.p.i.
untreated
2-DG
TNFα+IFNγ+IL-2+
2 cytokines
1 cytokine
none
n.s.
h
MC38OVA
CD45.1+
6 days
4 Gy
24 h
CD45.2+ OT-1 T cells
untreated or 2-DG treated
20 days
Tumor growth
Survival
i
untreated
2-DG treated
OCR:ECAR ratio
j
glycolytic ATP
mitochondrial ATP
33.1%
19.3%
untreated
2-DG treated
k
untreated
2-DG treated
norm. expression (gMFI)
PD-1
Tim-3
Lag3
TIGIT
Ly108
l
OT-1 transfer
no transfer
OT-1 untreated
OT-1 2-DG treated
Tumor area (mm2)
Time (days)

Supplementary Figure 9. HIF-1α-mediated glycolytic reprogramming drives terminal T cell differentiation. (a, b) Differentiation of WT and HIF-1α-deficient (*Hif1a*$^{fl/fl}$*Cd4*^{Cre}) T cells under hypoxia *in vitro*. Flow cytometric analyses of exhaustion and memory marker expression in WT and HIF-1α-deficient T cells, means ± SEM of 4-5 mice. **(c)** Limitation of glycolysis prevents the upregulation of co-inhibitory receptors on mPiC-deficient (*Slc25a3*$^{fl/fl}$*Cd4*^{Cre}) T cells *in vitro*. Flow cytometric analyses of Tim-3, granzyme A and Lag3 expression in WT and mPiC-deficient T cells treated with or without 2-desoxy-glucose (2-DG); means ± SEM of 3 mice. **(d-g)** Inhibition of glycolytic reprogramming sustains the stemness of virus-specific T cells *in vivo*. **(d)** Adoptive co-transfer of 2-DG pre-treated (tdTomato^{+} GFP^{+}) and control (GFP^{+}) P14 T cells into C57BL/6 mice after chronic infection with LCMV^{CL13} (as shown in Fig. 6h). **(e)** Flow cytometric analysis of P14 Tpex and Tex cells 21 d.p.i.; n = 6 mice. **(f)** Ratios of 2-DG pre-treated (tdTomato^{+}GFP^{+}) and control (GFP^{+}) donor P14 T cells in the spleens and LNs of recipient mice at days 10 and 21 after transfer; means ± SEM of 6 mice per timepoint. **(g)** Analysis of polyfunctional TNFα, IFNγ and IL-2 expression after PMA/iono re-stimulation of 2-DG-treated and control P14 T cells 21 days after co-transfer into chronically infected mice (as shown in Fig. 6h); means ± SEM of 6 mice. **(h-l)** Short-term 2-DG treatment augments the stemness and antitumor immunity of OT-1 T cells. **(h)** $1x10^{6}$ control or 2-DG-treated OT-1 T cells were adoptively transferred into irradiated C57BL/6 mice 7 days after MC38^{OVA} tumor inoculation. **(i)** Ratio of oxygen consumption rate (OCR) to extracellular acidification rate (ECAR) in control and 2-DG-treated T cells *in vitro*; means ± SEM of 3 mice. **(j)** Relative contribution of glycolysis and mitochondrial respiration to cellular ATP production in control and 2-DG-treated OT-1 T cells; means ± SEM of 3 mice. **(l)** Analysis of MC38^{OVA} growth after transfer of 2-DG-treated or control OT-1 T cells; means ± SEM of 4 mice. *, p<0.05; **, p<0.01; ***, p<0.001 by unpaired or paired Student's t-test in (a-c), (e), (g), (k) and (i). *, p<0.05 by 2-way ANOVA in (l).

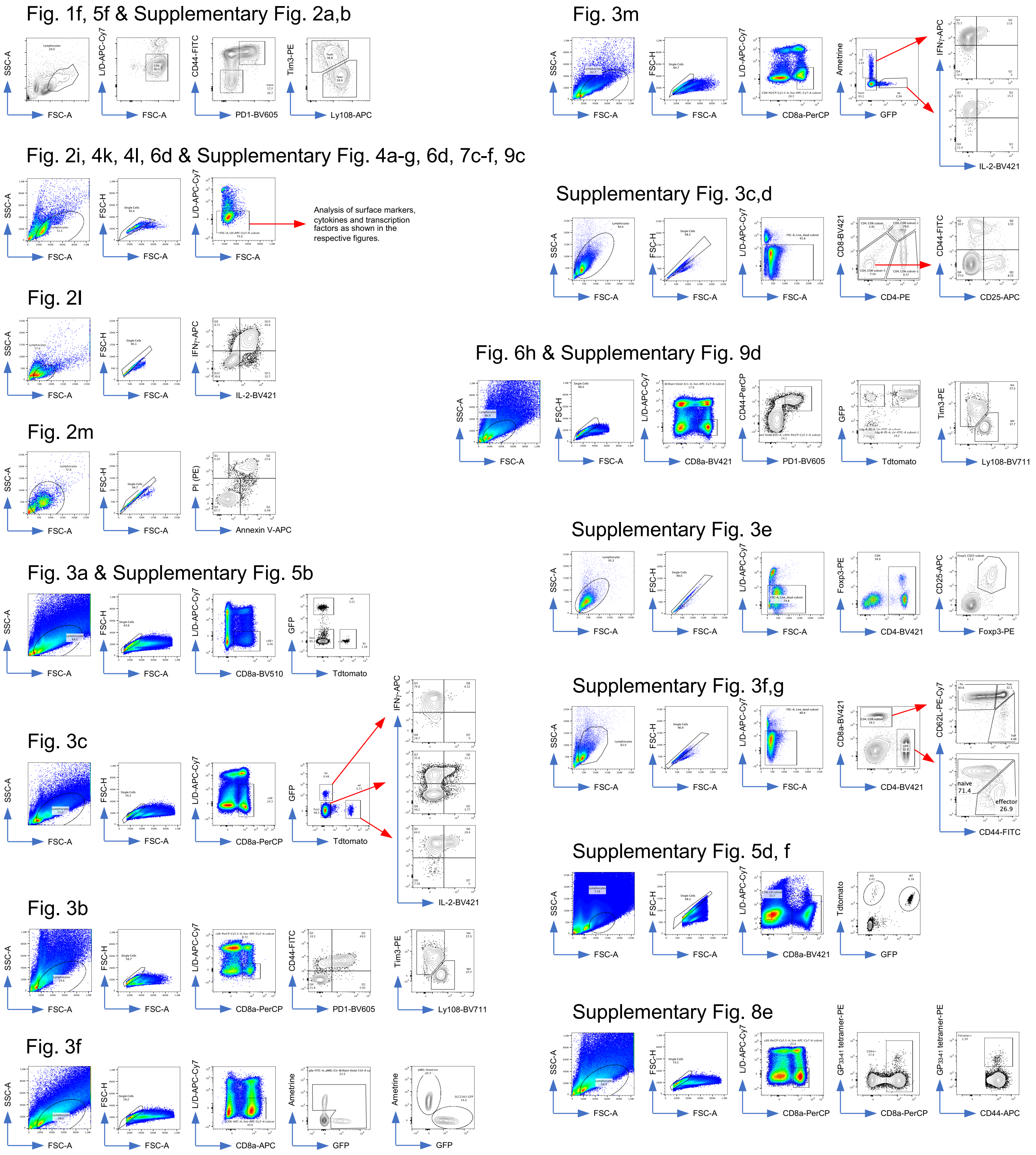

Supplementary Figure 10. Gating strategies for the flow cytometric detection of immune cells. Analysis of T cell subsets and markers by flow cytometry in the main and supplementary figures as indicated.

Supplementary Table S1. Gene sets used in this study.

Gene Set ID	Systematic gene set name	Reference / Source	Data in figure
M9577	MOOTHA_MITOCHONDRIA	Mootha *at al.* 2003 (PMID: 12808457)	Fig. 1e<br>Supplementary Fig. 1e<br>Supplementary Fig. 8d
M19046	MITOCHONDRIAL_RESPIRATORY_CHAIN	GO:0005746 (Gene ontology)	Fig. 1e<br>Supplementary Fig. 8d
M27446	REACTOME_MITOCHONDRIAL_TRANSLATION	R-HSA-5368287 (REACTOME)	Supplementary Fig. 1e
M1025	REACTOME_RESPIRATORY_ELECTRON_TRANSPORT_ATP_SYNTHESIS_BY_CHEMIOSMOTIC_COUPLING_AND_HEAT_PRODUCTION_BY_UNCOUPLING_PROTEINS	R-HSA-163200 (REACTOME)	Supplementary Fig. 1e
M19540	KEGG_OXIDATIVE_PHOSPHORYLATION	hsa00190 (Kyoto Encyclopedia of Genes and Genomes)	Fig. 2f
M5834	GSE9650_EFFECTOR_VS_EXHAUSTED_CD8_TCELL	GSE9650_1256_200_DN Wherry *et al.* 2007 (PMID: 17950003)	Fig. 2g
M5938	HALLMARK_REACTIVE_OXYGEN_SPECIES_PATHWAY	HALLMARK gene set Liberzon *et al.* 2025 (PMID: 26771021)	Fig. 4c
M1386	KEGG_PENTOSE_PHOSPHATE_PATHWAY	hsa00030 (Kyoto Encyclopedia of Genes and Genomes)	Supplementary Fig. 6b
M17905	ELVIDGE_HIF1A_TARGETS_UP	Elvidge *et al.* 2006 (PMID: 16565084)	Supplementary Fig. 6h
M2513	ELVIDGE_HIF1A_TARGETS_DN	Elvidge *et al.* 2006 (PMID: 16565084)	Supplementary Fig. 6h

Supplementary Table S2. Flow cytometry antibodies used in this study.

Antibody	Clone	Company	Cat #	RRID	Dilution
anti-CD4 (APC-Cy7-conjugated)	GK1.5	BD Biosciences	552501	AB_394331	1:400
anti-CD4 (PE-conjugated)	GK1.5	Biolegend	100408	AB_312693	1:400
anti-CD19 (APC-Cy7-conjugated)	1D3	BD Biosciences	557655	AB_396770	1:400
anti-CD25 (APC-conjugated)	PC61	Biolegend	102012	AB_312861	1:400
anti-CD69 (BV605-conjugated)	H1.2F3	Biolegend	104530	AB_2563062	1:400
anti-CD44 (PE-conjugated)	IM7	Thermo Fischer Scientific	12-0441-82	AB_465664	1:800
anti-CD44 (APC-conjugated)	IM7	Biolegend	103012	AB_312963	1:400
anti-CD44 (FITC conjugated)	IM7	Biolegend	103006	AB_312957	1:400
anti-CD8a (BV421-conjugated)	53-5.7	Biolegend	100753	AB_2562558	1:400
anti-CD8a (PerCP-Cy5.5)	53-5.7	Biolegend	100734	AB_2075238	1:400
anti-CD8a (APC-conjugated)	53-5.7	Biolegend	100712	AB_312751	1:400
anti-Tim3 (PE-conjugated)	RMT3-23	Biolegend	119704	AB_345378	1:400
anti-Tim3 (APC-conjugated)	RMT3-23	Biolegend	119705	AB_2561655	1:400
anti-Tim3 (PE-Cy7-conjugated)	RMT3-23	Biolegend	119716	AB_2571933	1:400
anti-Tim3 (BV711-conjugated)	RMT3-23	Biolegend	119727	AB_2716208	1:400
anti-Ly108 (APC-conjugated)	330-AJ	Biolegend	134610	AB_2728155	1:200
anti-Ly108 (BV711-conjugated)	13G3	BD Biosciences	740823	AB_2740481	1:200
anti-PD1 (BV605-conjugated)	29F.1A12	Biolegend	135219	AB_11125371	1:400
anti-CD62L (PerCP-Cy5.5)	MEL-14	Biolegend	104432	AB_2285839	1:200
anti-Granzyme A (PerCP-eF710)	3G8.5	Thermo Fischer Scientific	46-5831-82	AB_2573775	1:200
anti-Granzyme B (Alexa Fluor 700)	QA16A02	Biolegend	372222	AB_2728389	1:400
anti-Lag3 (BV650-conjugated)	C9B7W	Biolegend	125227	AB_2687209	1:400
anti-Foxp3 (PE-conjugated)	FJK-16s	Thermo Fischer Scientific	12-5773-82	AB_465936	1:200
anti-IFN-γ (APC-conjugated)	XMG1.2	Biolegend	505810	AB_315404	1:200
anti-IL-2 (BV421-conjugated)	JES6-5H4	Biolegend	503826	AB_2650897	1:200
anti-TNF-α (PE-Cy7-conjugated)	RMT3-23	Biolegend	119716	AB_2571933	1:200
anti-HIF-1α (Alexa Fluor 647)	546-16	Biolegend	359705	AB_2563331	1:100
anti-HIF-1α (HRP-conjugated)	D1S7W	Cell Signaling Technology	36169S	AB_2799095	1:1000
anti-HIF-1α (APC-conjugated)	Mgc3	Thermo Fischer Scientific	17-7528-82	AB_2802231	1:100
anti-CD4 (APC-Cy7-conjugated)	GK1.5	BD Biosciences	552051	AB_394331	1:400
anti-CD19 (APC-Cy7-conjugated)	1D3	BD Biosciences	557655	AB_396770	1:400
anti-MTCO1 (Alexa Fluor 488)	1D6E1A8	Abcam	ab198600	N/A	1:1000
anti-SDHA (Alexa Fluor 488)	2E3GC12FB 2AE2	Abcam	ab154473	N/A	1:1000
anti-SDHB (Alexa Fluor 488)	21A11AE7	Abcam	ab197902	AB_2889312	1:1000
anti-ATP5A1 (Alexa Fluor 488)	1B10H3	ProteinTech	CL488-66037	AB_2883234	1:1000
