# Supplementary material for: Mitochondrial dysfunction promotes the transition of precursor to terminally exhausted T cells through HIF-1α-mediated glycolytic reprogramming
Source: Nat Commun. 2023 Oct 27;14:6858. doi: 10.1038/s41467-023-42634-3 (PMC10611730; doi:10.1038/s41467-023-42634-3)
Supplement: Supplementary file 3 — Reporting Summary [file 41467_2023_42634_MOESM3_ESM.pdf]

nature portfolio

Corresponding author(s): Martin Vaeth

Last updated by author(s): Sep 27, 2023

Reporting Summary

Nature Portfolio wishes to improve the reproducibility of the work that we publish. This form provides structure for consistency and transparency in reporting. For further information on Nature Portfolio policies, see our Editorial Policies and the Editorial Policy Checklist.

Statistics

For all statistical analyses, confirm that the following items are present in the figure legend, table legend, main text, or Methods section.

n/a	Confirmed	
☐	☒	The exact sample size (*n*) for each experimental group/condition, given as a discrete number and unit of measurement
☐	☒	A statement on whether measurements were taken from distinct samples or whether the same sample was measured repeatedly
☐	☒	The statistical test(s) used AND whether they are one- or two-sided<br>*Only common tests should be described solely by name; describe more complex techniques in the Methods section.*
☒	☐	A description of all covariates tested
☒	☐	A description of any assumptions or corrections, such as tests of normality and adjustment for multiple comparisons
☐	☒	A full description of the statistical parameters including central tendency (e.g. means) or other basic estimates (e.g. regression coefficient) AND variation (e.g. standard deviation) or associated estimates of uncertainty (e.g. confidence intervals)
☒	☐	For null hypothesis testing, the test statistic (e.g. *F*, *t*, *r*) with confidence intervals, effect sizes, degrees of freedom and *P* value noted<br>*Give P values as exact values whenever suitable.*
☒	☐	For Bayesian analysis, information on the choice of priors and Markov chain Monte Carlo settings
☒	☐	For hierarchical and complex designs, identification of the appropriate level for tests and full reporting of outcomes
☒	☐	Estimates of effect sizes (e.g. Cohen's *d*, Pearson's *r*), indicating how they were calculated

Our web collection on statistics for biologists contains articles on many of the points above.

Software and code

Policy information about availability of computer code

Data collection	FACSDiva v9, SpectroFlo v2.2.0.2, Cell Ranger v3.0.2, Seahorse XFe96 Software package
Data analysis	Seurat R package v3.2, Slingshot 1.4.0, CellRanger software v2.0.2, STAR v2.7.2b, Cytoscape v3.10.1, FACSDiva v9, GraphPad Prism v8, MetaboAnalyst 4.0, EnrichR (online tool), GSEA 4.3.0, oPPSSUM, Seahorse XFe96 Extracellular Flux Analyzer Software package, BioRender

For manuscripts utilizing custom algorithms or software that are central to the research but not yet described in published literature, software must be made available to editors and reviewers. We strongly encourage code deposition in a community repository (e.g. GitHub). See the Nature Portfolio guidelines for submitting code & software for further information.

Data

Policy information about availability of data

All manuscripts must include a data availability statement. This statement should provide the following information, where applicable:

- Accession codes, unique identifiers, or web links for publicly available datasets
- A description of any restrictions on data availability
- For clinical datasets or third party data, please ensure that the statement adheres to our policy

The bulk and single cell RNA sequencing data generated in this study have been deposited in the Gene Expression Omnibus (GEO) database under accession codes GSE212298 (https://www.ncbi.nlm.nih.gov/geo/query/acc.cgi?acc=GSM6523603), GSE214003 https://www.ncbi.nlm.nih.gov/geo/query/acc.cgi?acc=GSE214003), and GSE213847 (https://www.ncbi.nlm.nih.gov/geo/query/acc.cgi?acc=GSE213847). The remaining data are available within the Article, Supplementary Information

or Source Data file. Source data are provided with this paper.

Human research participants

Policy information about studies involving human research participants and Sex and Gender in Research.

Reporting on sex and gender	N/A
Population characteristics	N/A
Recruitment	N/A
Ethics oversight	N/A

Note that full information on the approval of the study protocol must also be provided in the manuscript.

Field-specific reporting

Please select the one below that is the best fit for your research. If you are not sure, read the appropriate sections before making your selection.

[x] Life sciences [ ] Behavioural & social sciences [ ] Ecological, evolutionary & environmental sciences

For a reference copy of the document with all sections, see nature.com/documents/nr-reporting-summary-flat.pdf

Life sciences study design

All studies must disclose on these points even when the disclosure is negative.

Sample size	No statistical methods were used to pre-determine sample sizes but our sample sizes are similar to those reported in previous publications (Vaeth et al., Immunity 2016 and Hochrein et all Cell Metabolism 2022). For all in vivo and in vitro experiments, minimum 3 biological replicates and minimum 2 independent experiments were performed. Samples size and number of independent experiments are indicated in the figure legends.
Data exclusions	No data were excluded from analysis.
Replication	All experiments were repeated (> 2 times) and reproducible. scRNA-Seq was not repeated, but validated by FACS analysis.
Randomization	Randomization was not relevant to this study. Mice were grouped by genotype/treatment and treated equally.
Blinding	No blinding was performed as mice were grouped by genotype/and treated equally. No human subjects were investigated.

Behavioural & social sciences study design

All studies must disclose on these points even when the disclosure is negative.

Study description	N/A
Research sample	N/A
Sampling strategy	N/A
Data collection	N/A
Timing	N/A
Data exclusions	N/A
Non-participation	N/A
Randomization	N/A

Ecological, evolutionary & environmental sciences study design

All studies must disclose on these points even when the disclosure is negative.

Study description	N/A
Research sample	N/A
Sampling strategy	N/A
Data collection	N/A
Timing and spatial scale	N/A
Data exclusions	N/A
Reproducibility	N/A
Randomization	N/A
Blinding	N/A

Did the study involve field work? ☐ Yes ☒ No

Reporting for specific materials, systems and methods

We require information from authors about some types of materials, experimental systems and methods used in many studies. Here, indicate whether each material, system or method listed is relevant to your study. If you are not sure if a list item applies to your research, read the appropriate section before selecting a response.

Materials & experimental systems

n/a	Involved in the study	
☐	☒	Antibodies
☐	☒	Eukaryotic cell lines
☒	☐	Palaeontology and archaeology
☐	☒	Animals and other organisms
☒	☐	Clinical data
☒	☐	Dual use research of concern

Methods

n/a	Involved in the study	
☒	☐	ChIP-seq
☐	☒	Flow cytometry
☒	☐	MRI-based neuroimaging

Antibodies

Antibodies used

Goat polyclonal anti-hamster IgG, MP Biomedicals,Cat#: 56984 1:10000
Hamster monoclonal anti-mouse CD3, Bio X Cell, Cat#: BE0001-1; Clone 145-2C1 (0.5ug/ml)
Hamster monoclonal anti-mouse CD28, Bio X Cell,Cat#: BE00015-1; Clone 37.51 (1ug/ml)
anti-FcgRII/FcgRIII antibody ,Bio X Cell, Cat#: BE0307; Clone 2.4G2 1:200
Rat anti-CD4 (in vivo depletion), Bio X Cell, Cat# BE0003-1; Clone GK1.5
Rat anti-CD44(FITC-conjugated), Biolegend Cat#: 103006; Clone IM7 1:400
Rat anti-CD4(APC-Cy7-conjugated), BD Biosciences, Cat#: 552501; Clone GK1.5 1:400
Rat anti-CD19(APC-Cy7-conjugated), BD Biosciences, Cat#: 557655; Clone 1D3 1:400
Rat anti-CD44(PE-conjugated), Thermo Fischer Scientific, Cat#: 12-0441-82; Clone IM7 1:400
Rat anti-CD44(APC-conjugated), Biolegend, Cat#: 103012; Clone IM7 1:400
Rat anti-CD8a(BV421-conjugated), Biolegend, Cat#: 100753; Clone 53-5.7 1:400
Rat anti-CD8a (PerCP-Cy5.5-conjugated), Biolegend, Cat#: 100734; Clone 53-5.7 1:400
Rat anti-CD8a(APC-conjugated), Biolegend, Cat#: 100712; Clone 53-5.7 1:400
Rat anti-Tim3(PE-conjugated), Biolegend, Cat#: 119704; Clone RMT3-23 1:400
Rat anti-Tim3(APC-conjugated), Biolegend, Cat#: 119705; Clone RMT3-23 1:400
Rat anti-Tim3(PE-Cy7-conjugated), Biolegend, Cat#: 119716; Clone RMT3-23 1:400
Rat anti-Tim3(BV711-conjugated), Biolegend, Cat#: 119727; Clone RMT3-23 1:400
Mouse anti-Slamf6(Ly108) (APC-conjugated), Biolegend, Cat#: 134610; Clone 330-AJ 1:200
Mouse anti- Slamf6(Ly108) (BV711-conjugated), BD Biosciences, Cat#: 740823; Clone 13G3 1:200
Rat anti-PD1(BV605-conjugated), Biolegend, Cat#: 135219; Clone 29F.1A12 1:400

Rat anti-CD62L (PerCP-Cy5.5-conjugated), Biolegend, Cat#: 104432; Clone MEL-14 1:200
Rat anti-Granzyme A (PerCP-eF710-conjugated), Thermo Fischer Scientific, Cat#: 46-5831-82; Clone 3G8.5 1:200
Rat anti-Lag3 (BV650-conjugated) Biolegend Cat#: 125227; Clone C9B7W 1:200
Rat anti-IFN-g (APC-conjugated) Biolegend Cat#: 505810; Clone XMG1.2 1:200
Rat anti-IL-2 (BV421-conjugated) Biolegend Cat#: 503826; Clone JES6-5H4 1:200
Rat anti-TNF-a(PE-Cy7-conjugated) Biolegend Cat#: 119716; Clone RMT3-23 1:200
Mouse anti- HIF-1a (Alexa Fluor 647-conjugated) Biolegend Cat#: 359705; Clone 546-16 1:100
Rabbit anti-HIF-1a (HRP-conjugated) Cell Signaling Cat#: 36169S; Clone D1S7W 1:1000
Goat anti-Rabbit-HRP Bio-Rad Cat#: STAR208P; Polyclonal 1:2000
Mouse anti-beta-actin-HRP Biolegend Cat#: 643807; Clone 2F1-1 1:5000
Mouse anti- HIF-1a (APC-conjugated) Thermo Fischer Scientific Cat#: 17-7528-82; Clone Mgc3 1:100
Rat anti-CD4(APC-Cy7-conjugated) BD Biosciences Cat#: 552051; Clone GK1.5 1:400
TotalSeq™-A0837 anti-mouse IL-33Rα (IL1RL1, ST2) Biolegend Cat#: 145317; Clone DIH9 1:400
TotalSeq™-A0250 anti-mouse/human KLRG1 (MAFA) Biolegend Cat#: 138431; Clone 2F1/KLRG1 1:400
TotalSeq™-A0846 anti-mouse CD185 (CXCR5) Biolegend Cat#: 145535; Clone L138D7 1:400
TotalSeq™-A0847 anti-mouse CD278 (ICOS) Biolegend Cat#: 117409; Clone 7E.17G9 1:400
TotalSeq™-A0201 anti-mouse CD103 Biolegend Cat#: 121437; Clone 2E7 1:400
TotalSeq™-A0421 anti-mouse CD49b Biolegend Cat#: 103523; Clone HMα2 1:400
TotalSeq™-C0595 anti-mouse CD11a Antibody Biolegend Cat#: 101131; Clone M17/4 1:400
TotalSeq™-A0191 anti-mouse/rat/human CD27 Biolegend Cat#: 124235; Clone LG.3A10 1:400
TotalSeq™-A0198 anti-mouse CD127 (IL-7Rα) Biolegend Cat#: 135045; Clone A7R34 1:400
TotalSeq™-A0807 anti-mouse CD200R (OX2R) Biolegend Cat#: 123913; Clone OX-100 1:400
TotalSeq™-A0301 anti-mouse Hashtag 1 Biolegend Cat#: 155801; Clone M1/42; 30-F11 1:400
TotalSeq™-A0302 anti-mouse Hashtag 2 Biolegend Cat#: 155803; Clone M1/42; 30-F11 1:400
Mouse anti-SDHA(Alexa Fluor 488 conjugated), Abcam, Cat#: ab154473; Clone 2E3GC12FB2AE2 1:1000
Mouse anti-SDHB(Alexa Fluor 488 conjugated), Abcam, Cat#: ab197902; Clone 21A11AE7 1:1000
Mouse anti-ATP5A1(CoraLite Plus 488 conjugated), ProteinTech Cat#: CLL488-66037; Clone 1B10H3 1:1000
Mouse anti-MTCOI(Alexa Fluor 647 conjugated), Abcam, Cat#: ab198600; Clone 1D6E1A8 1:1000
Rabbit anti-PGC-1a (unconjugated), ThermoFisher, Cat#:PA5-72948; Polyclonal 1:1000
Rabbit anti-TFAM (unconjugated), ThermoFisher, Cat#:PA5-29571; Polyclonal 1:1000

Validation

All antibodies used are commercially available and have been validated by the manufacturers.

BioLegend:
https://www.biolegend.com/en-us/quality/quality-control
Specificity testing of 1-3 target cell types with either single- or multi-color analysis (including positive and negative cell types).
Once specificity is confirmed, each new lot must perform with similar intensity to the in-date reference lot. Brightness (MFI) is evaluated from both positive and negative populations.
Each lot product is validated by QC testing with a series of titration dilutions.

Abcam:
https://www.abcam.com/en-it/products/primary-antibodies/alexa-fluor-488-anti-sdha-antibody-epr9043b-ab309691
https://www.abcam.com/products/primary-antibodies/alexa-fluor-488-sdhb-antibody-21a11ae7-ab197902.html
https://www.abcam.com/products/primary-antibodies/alexa-fluor-647-mtco1-antibody-1d6e1a8-ab198600.html

Cell Signalling:
https://www.cellsignal.com/products/primary-antibodies/hif-1a-d1s7w-xp-rabbit-mab/36169

BD:
https://www.bdbiosciences.com/en-de/products/reagents/flow-cytometry-reagents/research-reagents/single-color-antibodies-ruo/apc-cy-7-rat-anti-mouse-cd19.557655
https://www.bdbiosciences.com/en-us/products/reagents/flow-cytometry-reagents/research-reagents/single-color-antibodies-ruo/bv711-mouse-anti-mouse-ly-108.740823
https://www.bdbiosciences.com/en-pl/products/reagents/flow-cytometry-reagents/research-reagents/single-color-antibodies-ruo/apc-cy-7-rat-anti-mouse-cd4.552051

eBioscience / Invitrogen / Thermo Fisher:
https://www.thermofisher.com/antibody/product/CD44-Antibody-clone-IM7-Monoclonal/12-0441-82
https://www.thermofisher.com/antibody/product/Granzyme-A-Antibody-clone-GzA-3G8-5-Monoclonal/46-5831-82
https://www.thermofisher.com/antibody/product/HIF-1-alpha-Antibody-clone-Mgc3-Monoclonal/17-7528-82
https://www.thermofisher.com/antibody/product/PGC1-alpha-Antibody-Polyclonal/PA5-72948
https://www.thermofisher.com/antibody/product/TFAM-Antibody-Polyclonal/PA5-29571

Bio-Rad ：
https://www.bio-rad-antibodies.com/polyclonal/rabbit-lapine-igg-antibody-star208.html?f=hrp

ProteinTech:
https://www.ptglab.com/products/ATP5A1-Antibody-CL488-66037.htm

Eukaryotic cell lines

Policy information about cell lines and Sex and Gender in Research

Cell line source(s)	Platinum-E retroviral packaging cell line was bought from Cell Biolabs Inc. (Cat# RV-101). The MC38-OVA and B16F10-OVA cell lines were kind gifts from DR. Edgar Serfling (University of Würzburg) and DR. Tobias Bald (University of Bonn).
Authentication	No specific authentication of the cell lines was performed.
Mycoplasma contamination	Cell lines were only used for experiments if tested negative for mycoplasma contaminations.
Commonly misidentified lines (See ICLAC register)	No commonly misidentified lines were used in this study.

Palaeontology and Archaeology

Specimen provenance	N/A
Specimen deposition	N/A
Dating methods	N/A

☐ Tick this box to confirm that the raw and calibrated dates are available in the paper or in Supplementary Information.

Ethics oversight	*Identify the organization(s) that approved or provided guidance on the study protocol, OR state that no ethical approval or guidance was required and explain why not.*

Note that full information on the approval of the study protocol must also be provided in the manuscript.

Animals and other research organisms

Policy information about studies involving animals; ARRIVE guidelines recommended for reporting animal research, and Sex and Gender in Research

Laboratory animals	All mice were bred and maintained under specific pathogen free (SPF) conditions in the Center for Experimental Medicine (ZEMM) or the Institute for Systems Immunology at the Julius-Maximilians University of Würzburg. Mice were maintained on a 12/12 h light/dark cycle at between 20-24 °C in individually ventilated cages. Mice had access to standard chow (Ssniff; cat# V1534) and autoclaved water ad libitum and health status of the animals was inspected daily by the responsible caretakers. Hygiene status of the sentinel mice was monitored quarterly according to the FELASA guidelines. Both male and female mice between 8 and 24 weeks of age at the time of the experiment were used for the in vitro experiments described in this study. For chronic LCMV infection models, male mice between 8 and 12 weeks of age were used.<br><br>Strains: C57BL/6 (JAX strain 000664), CD45.1+ (strain 002014), P14 (strain 037394), OT-1 (strain 003831), Ubi-GFP (strain 004353), tdTomato (Kastenmüller et al.), mito-Dendra2 (strain 018397), Hif1afl/fl (strain 007561), Tfamfl/fl (strain 026123), Rag1–/– (strain 002216) and Cd4Cre mice (strain 017336) were purchased from the Jackson Laboratories (JAX) and/or maintained at our institution. Slc25a3fl/fl (mPiC) mice and Nfatc1fl/fl mice were kindly provided by Jeffery D. Molkentin (Cincinnati Children's Hospital Medical Center, OH, USA) and Anjana Rao (La Jolla Institute for Immunology, CA, USA). All animals used in this study were on a C57BL/6 genetic background.
Wild animals	No wild animals were used in this study
Reporting on sex	Both male and female mice between 8 and 24 weeks of age at the time of the experiment were used for the in vitro experiments described in this study. For chronic LCMV infection models, male mice between 8 and 12 weeks of age were used.
Field-collected samples	No field-collected samples were used in this study.
Ethics oversight	All protocols used for animal experimentation were approved by the animal ethics/welfare committee of the government of Lower Franconia, Germany

Note that full information on the approval of the study protocol must also be provided in the manuscript.

Clinical data

Policy information about clinical studies

All manuscripts should comply with the ICMJEguidelines for publication of clinical research and a completedCONSORT checklist must be included with all submissions.

Clinical trial registration	N/A
Study protocol	N/A

Data collection	N/A
Outcomes	N/A

Dual use research of concern

Policy information about dual use research of concern

Hazards

Could the accidental, deliberate or reckless misuse of agents or technologies generated in the work, or the application of information presented in the manuscript, pose a threat to:

No	Yes	
☒	☐	Public health
☒	☐	National security
☒	☐	Crops and/or livestock
☒	☐	Ecosystems
☒	☐	Any other significant area

Experiments of concern

Does the work involve any of these experiments of concern:

No	Yes	
☒	☐	Demonstrate how to render a vaccine ineffective
☒	☐	Confer resistance to therapeutically useful antibiotics or antiviral agents
☒	☐	Enhance the virulence of a pathogen or render a nonpathogen virulent
☒	☐	Increase transmissibility of a pathogen
☒	☐	Alter the host range of a pathogen
☒	☐	Enable evasion of diagnostic/detection modalities
☒	☐	Enable the weaponization of a biological agent or toxin
☒	☐	Any other potentially harmful combination of experiments and agents

ChIP-seq

Data deposition

☐ Confirm that both raw and final processed data have been deposited in a public database such as GEO.

☐ Confirm that you have deposited or provided access to graph files (e.g. BED files) for the called peaks.

Data access links *May remain private before publication.*	N/A
Files in database submission	N/A
Genome browser session (e.g. UCSC)	N/A

Methodology

Replicates	N/A
Sequencing depth	N/A
Antibodies	N/A
Peak calling parameters	N/A
Data quality	N/A
Software	N/A

Flow Cytometry

Plots

Confirm that:

- [x] The axis labels state the marker and fluorochrome used (e.g. CD4-FITC).
- [x] The axis scales are clearly visible. Include numbers along axes only for bottom left plot of group (a 'group' is an analysis of identical markers).
- [x] All plots are contour plots with outliers or pseudocolor plots.
- [x] A numerical value for number of cells or percentage (with statistics) is provided.

Methodology

Sample preparation	A single cell suspension was prepared from the spleen and lymph nodes of donor mice. Red blood cell were lysed by lysis buffer before samples were used for CD8 T cell isolation or FACS analysis.
Instrument	Data was collected on a BD FACSCelesta or Attune flow cytometers. Cell sorting was done using a BD FACSAria III.
Software	BD Flowjo (version 10.7.2) was used for all analysis
Cell population abundance	Post Sort analysis was routinely performed on sorted samples and the target population was above 95% total events and about 100% of cells in a FSC-A/SSC-A lymphocyte gate.
Gating strategy	Singlets were gated using FSC-H/FSC-A and then SSC-A/SSC-H. Dead cells were labeled by a viability staining.

- [x] Tick this box to confirm that a figure exemplifying the gating strategy is provided in the Supplementary Information.

Magnetic resonance imaging

Experimental design

Design type	N/A
Design specifications	N/A
Behavioral performance measures	N/A

Acquisition

Imaging type(s)	N/A
Field strength	N/A
Sequence & imaging parameters	N/A
Area of acquisition	N/A

Diffusion MRI ☐ Used ☐ Not used

Preprocessing

Preprocessing software	N/A
Normalization	N/A
Normalization template	N/A
Noise and artifact removal	N/A
Volume censoring	N/A

Statistical modeling & inference

Model type and settings	N/A
Effect(s) tested	N/A

Specify type of analysis: ☐ Whole brain ☐ ROI-based ☐ Both

Statistic type for inference (See Eklund et al. 2016)	N/A
Correction	N/A

Models & analysis

n/a	Involved in the study
☒	☐ Functional and/or effective connectivity
☒	☐ Graph analysis
☒	☐ Multivariate modeling or predictive analysis
